# Multifunctional Porous Hydrogen-Bonded Organic Frameworks: Current Status and Future Perspectives

**DOI:** 10.1021/acscentsci.2c01196

**Published:** 2022-12-16

**Authors:** Zu-Jin Lin, Shaheer A. R. Mahammed, Tian-Fu Liu, Rong Cao

**Affiliations:** † State Key Laboratory of Structural Chemistry, Fujian Institute of Research on the Structure of Matter, Chinese Academy of Sciences, Fuzhou 350002, P. R. China; ‡ College of Life Science, Fujian Agriculture and Forestry University, Fuzhou, Fujian 350002, P. R. China; § Fujian Science & Technology Innovation Laboratory for Optoelectronic Information of China, Fuzhou, Fujian 350108, P. R. China

## Abstract

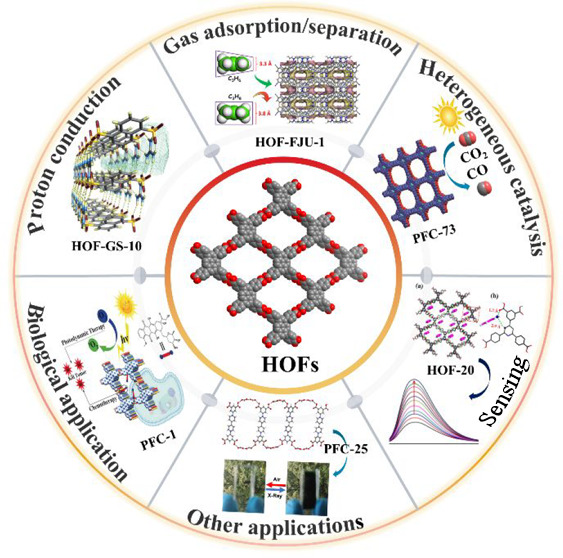

Hydrogen-bonded organic frameworks (HOFs), self-assembled from organic or metalated organic building blocks (also termed as tectons) by hydrogen bonding, π–π stacking, and other intermolecular interactions, have become an emerging class of multifunctional porous materials. So far, a library of HOFs with high porosity has been synthesized based on versatile tectons and supramolecular synthons. Benefiting from the flexibility and reversibility of H-bonds, HOFs feature high structural flexibility, mild synthetic reaction, excellent solution processability, facile healing, easy regeneration, and good recyclability. However, the flexible and reversible nature of H-bonds makes most HOFs suffer from poor structural designability and low framework stability. In this Outlook, we first describe the development and structural features of HOFs and summarize the design principles of HOFs and strategies to enhance their stability. Second, we highlight the state-of-the-art development of HOFs for diverse applications, including gas storage and separation, heterogeneous catalysis, biological applications, sensing, proton conduction, and other applications. Finally, current challenges and future perspectives are discussed.

## Abstract 2

HOFs are emerging as a unique type of multifunctional porous material. Herein, we summarize the design principles and various applications of HOFs as well as the prospect of their future development.

## 1 Introduction

Porous materials have been widely utilized in modern industry, being indispensable in our daily life. In the last three decades, great progress has been achieved in a special type of porous materials that have order structures and contain organic components, which can be divided into metal–organic frameworks (MOFs, also known as porous coordination polymers), covalent organic frameworks (COFs), and hydrogen-bonded organic frameworks (HOFs). They are usually constructed by predesigned organic building blocks and preconceived topologies. Therefore, these materials have much richer structural diversity, higher structural flexibility, and larger structural tunability than traditional inorganic porous materials like zeolites and porous carbons. Through either one-pot synthesis or postsynthesis, the porosity, surface area, pore size and shape, and even the chemical functionality of the MOFs/COFs/HOFs can be tailored for various applications, such as gas storage and separation, catalysis, drug delivery, sensing, etc.

HOFs are often prepared by self-assembling elegantly predesigned organic or metalated organic building blocks (also termed tectons) via hydrogen bonding, π–π packing, and other intermolecular interactions. The concept of HOFs was coined by Chen et al. in 2011.^1^ They reported the first HOF (i.e., HOF-1) with permanent porosity, which was used as a solid absorbent for highly selective adsorptive separation of C_2_H_2_ and C_2_H_4_ at room temperature. Interestingly, many scientists had started to investigate this type of material even before the introduction of the HOF concept. In 1969, Duchamp and Marsh isolated a 2D honeycomb hydrogen-bonded network that was self-assembled by benzene-1,3,5-tricarboxylic acid (TMA).^2^ No voids could be observed in this material because of the catenation in the 2D network. In 1987, Herbstein et al. used long-chain alkanes and primary alcohols as a template to prepare noncatenated hydrogen-bonded networks based on TMA, in which 1D channels are filled with long-chain template molecules.^3^ In 1988, Ermer reported a 3D hydrogen-bonded network based on adamantane-1,3,5,7-tetracarboxylic acid (ADTA), which is nonporous due to the 5-fold interpenetration of the *dia* network.^4^ In 1991, Ermer and Wuest independently synthesized 3D hydrogen-bonded networks with small guest molecules filled in their channels.^5,6^ Subsequently, a series of guest-inclusion hydrogen-bonded networks with 1D to 3D architectures was successively reported.^7^ Indeed, these early studies largely promote the evolution and progress of constructing porous HOFs. However, the permanent porosity of hydrogen-bonded networks was not established until 2010.^8^ The permanent porosity establishment that came with the introduction of the HOF concept played an important role in propelling the HOF development. Since then, emphasis has been put on how to prepare porous HOFs and how to exploit their functionalities. Nowadays, HOFs have become a unique type of porous multifunctional material for various applications.^9−11^

Although substantial progress has been achieved, the development of HOFs still largely lags behind those of their MOF and COF counterparts. On one hand, the interactions between adjacent building blocks in HOFs are mainly H-bonds, whose bonding energy (e.g., 10–40 kJ mol^–1^ for H-bonds) is much smaller than those of coordination bonds (90–350 kJ mol^–1^) in MOFs and covalent bonds in COFs (300–600 kJ mol^–1^).^12^ As a result, most HOFs suffer from framework instability, which cannot retain their porous frameworks after removing guest solvents from their voids. On the other hand, H-bonds are more flexible, reversible, and less directional as compared to coordination and covalent bonds, making HOFs more challenging to predict and design precisely. A slight change in tectons may significantly alter HOF structures and properties. Additionally, polymorphism resulting from different linkages or distinct degrees of interpenetration/catenation also easily occurs during the self-assembly of HOFs. That is, dramatic structural change may be observed by only a subtle change in self-assembled conditions. For example, the self-assembly of 4,4′,4″-(1,3,5-triazine-2,4,6-triyl)tribenzoic acid (H_3_TATB) in three different solvents can yield three HOFs termed PFC-11–13, which have the same linkages yet very distinct structures and properties because of the different degrees of catenation.^13^

Just as one coin has two sides, hydrogen bond’s weakness, flexibility, and reversibility endow HOFs with unique features, including mild preparation, easy regeneration and recyclability, good solution processability, feasible self-healing, high biocompatibility, etc. Because of their weakness and reversibility, HOFs can be synthesized in high crystallinity under mild conditions. Besides, HOFs can be easily dissociated into pristine organic building frameworks in some solvents. In turn, the resulting solvents may form HOFs after the removal of volatile solvents by volatilization. Such a reversible process facilitates HOFs for solution processability, regeneration, and recyclability. H-bonding interactions are flexible and reversible, which may help the injured HOFs to recover their structures and thus enable HOFs to serve as seal-healing materials. In addition, compared with their MOF counterpart, most HOFs are metal-free, avoiding the cytotoxicity of metal ions. The above-mentioned advantages make HOFs an ideal platform for constructing multifunctional materials.

So far, some rules for structural design and stability enhancement for HOFs have been proposed. In this context, a growing number of robust HOFs with permanent porosity has been synthesized, and HOFs may also be considered as raw precursors that can be combined with other functional materials to fabricate multifunctional composite materials. Of late, some excellent reviews about the synthesis and applications of HOFs were also reported.^14−23^ In this Outlook, we would like to summarize the basic principles for the design and construction of robust HOFs. Emphasis was put on the discussion about recent impressive progress in the application of HOFs. Finally, we discuss some major opportunities and challenges for HOF-based multifunctional materials.

## 2 Structural Design of HOFs

Although many HOFs are synthesized by serendipity, the design of HOFs is a long-term pursuit of researchers. Reticular chemistry, which utilizes the geometry-guided design of periodic materials by the connection of well-defined building blocks through strong bonds, has been extensively employed to predict and design crystalline materials.^24^ To use this strategy, readily accessible building blocks with special geometries are essential, in addition to the other essential requirements called isoreticular chemistry, which enables the modification, replacement, expansion, and contraction of building blocks to customize the structural properties of periodic materials for different properties. Due to the discovery of plenty of well-defined metal-containing clusters (or metal ions) termed secondary building units (SBUs) and the easy accessibly of many robust organic ligands, reticular chemistry has proven to be an extremely effective platform for designing and predicting MOFs. Such a design method also can be directly transplanted to COFs because of the ready accessibility of well-defined building blocks of COFs, significantly spurring COF development.^25^

Reticular chemistry, however, is not widely applicable for the accurate prediction and design of HOFs. Although tectons with well-defined geometries are readily accessible, the connection linkages between tectons in HOFs, are always chaotic and unpredictable. The weak interaction combined with the great flexibility and low directionality of hydrogen bonds jointly contribute to the difficulty in obtaining fixed and robust linkages with a specific diagram during HOF synthesis. The instabilities and fragilities of linkages, as well as the interpenetration and catenation, significantly obstruct the design of HOFs by reticular chemistry.^25^

**Figure 1:**
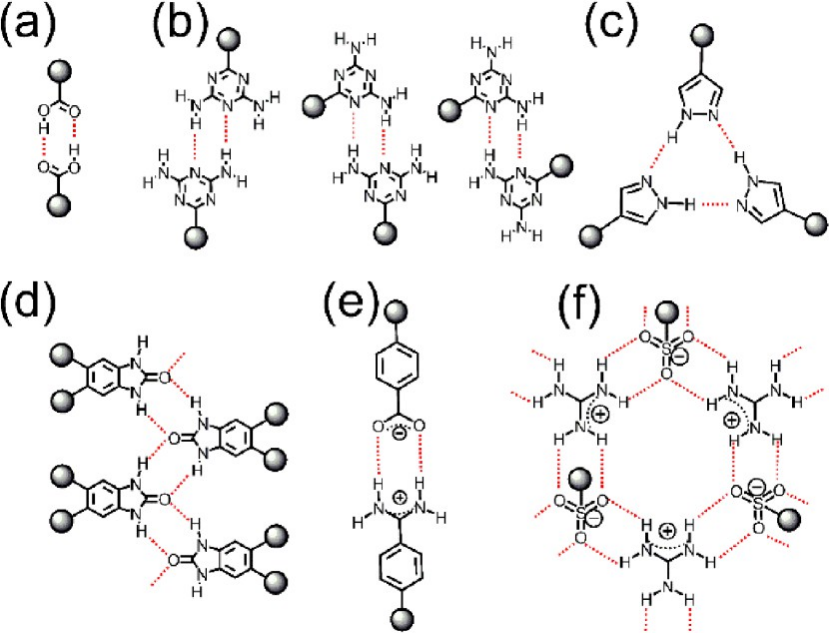
Supramolecular synthons most used for the construction of porous HOFs: (a) carboxyl dimer, (b) DAT dimer, (c) cyclic pyrazolyl trimer, (d) benzimidazolone chain, (e) amidinium-carboxylate, and (f) guanidinium-sulfonate.

**Figure 2:**
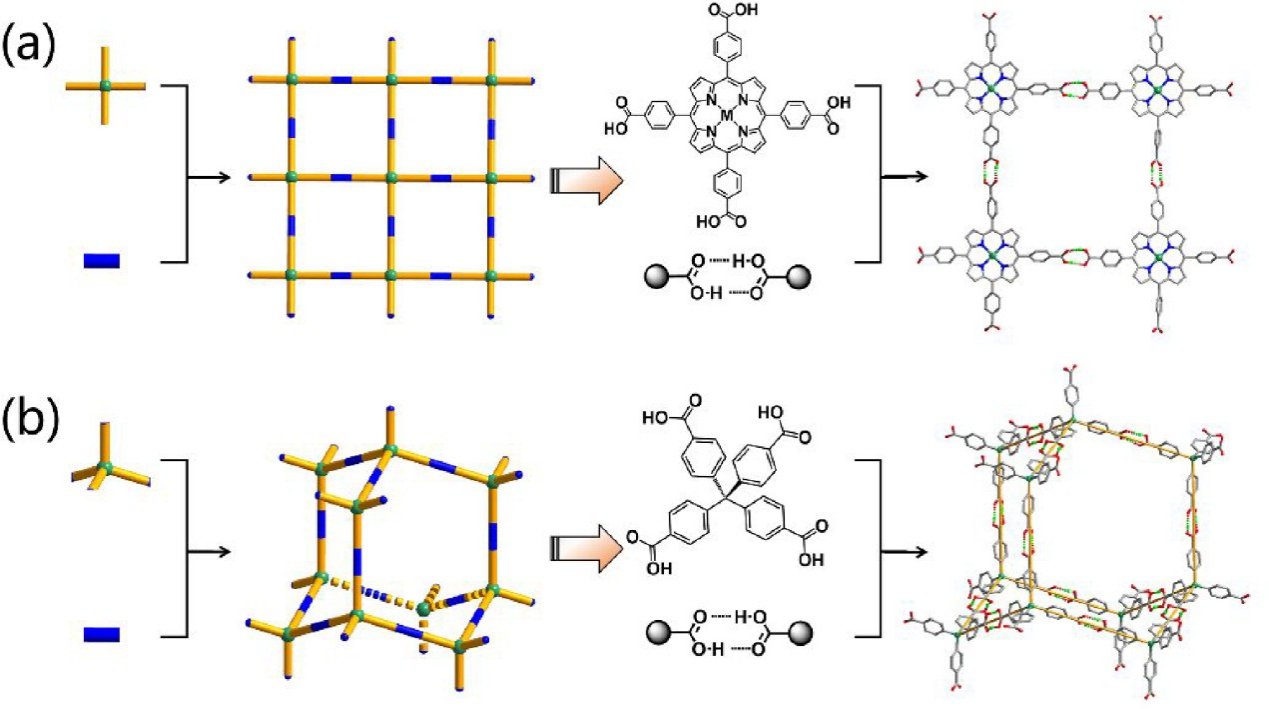
Examples show the reticular chemistry used for the design of HOFs with (a) *sql* and (b) *dia* topology, respectively.

Fortunately, scientists have explored supramolecular synthons that formed by multiple H-bonding pairs. Some of them have high directionality and exhibit a special spatial geometry, which can serve as relatively stable linkages for HOF design by reticular chemistry. As shown in Figure 1, carboxyl dimer, 2,4-daiminotriazine (DAT) dimer, cyclic pyrazolyl trimer, benzimidazolone chain, amidinium-carboxylate, and guanidinium-sulfonate are the most common supramolecular synthons used for the building of HOFs. Figure 2 shows typical examples for HOF design by reticular chemistry. So far, a library of stable HOFs with permanent porosity was successfully designed and synthesized by the collaborative choice of the above supramolecular synthons and rigid tectons.^14,22,23,26^ Some HOFs are so stable that their structures can be well retained even in harsh conditions like extremely acidic or basic aqueous solutions.

**Figure 3:**
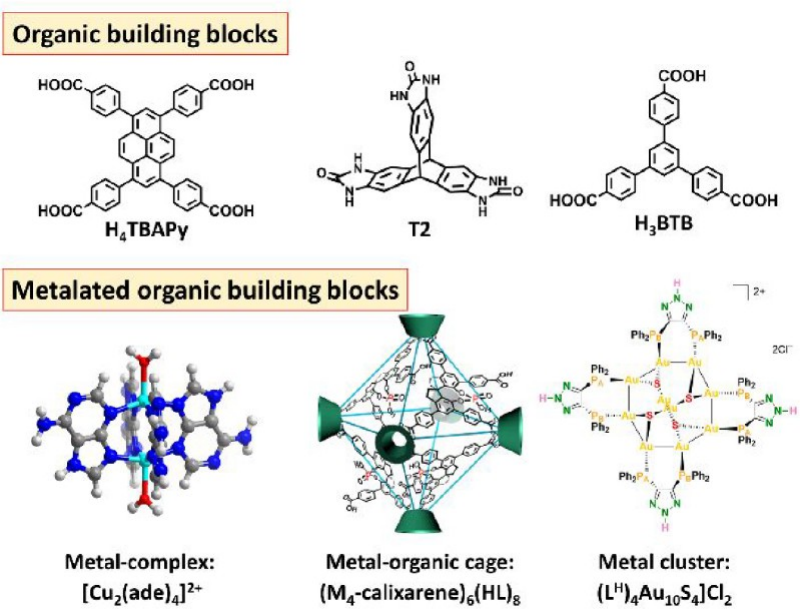
Representative tectons including pure organic building blocks and metalated organic building blocks used to construct HOFs. The pictures of the octahedral cage and clusters are reproduced with permission from refs (32) and (33), respectively. Copyright 2019 Nature Publishing Group and 2021 American Chemical Society.

Besides organic tectons, it is should be noted that metalated organic building blocks also can be utilized as tectons for the design and synthesis of HOFs (Figure 3). Metal complexes are one of the representative metalated organic building blocks that are most commonly employed for HOF construction. For example, a paddle-wheel metal complex [Cu_2_(ade)_4_] (ade = adenine, Figure 3), formed by the coordination of copper ions with adenines, is demonstrated to be a very good tecton to build microporous HOFs. Based on this tecton, several HOFs including SMOF-1/SMOF-2,^27^ MPM-1-TiFSIX,^28^ HOF-21,^29^ HOF-ZJU-101/HOF-ZJU-102,^30^ and SMOF-PFSIX-1/SMOF-AsFSIX-1^31^ have been prepared, which exhibited very good performances in gas adsorption and separation. Metal–organic cages also can serve as metalated organic building blocks for the preparation of HOFs. Gong et al. reported that the self-assembly of chiral 4,4′,6,6′-tetrakis(4-benzoic acid)-1,1′-spinol phosphonate (H_4_L) and M_4_-calixarene could isolate metal–organic-based octahedral cages (Figure 3), which further connected to each other through H-bonds to form 3D HOFs.^32^ Besides, some metal–organic clusters bearing hydrogen donors and acceptors can also be employed as metalated organic building blocks for HOF design and preparation. For instance, Yam et al. successfully isolated a cluster-based HOF L^H^-Au_10_S_4_-Cl [L^H^ = 4,5-bis(diphenylphosphanyl)-2*H*-1,2,3-triazole] based on a decanuclear gold(I)-sulfido cluster (Figure 3), which is capable of separating benzene/cyclohexane accompanied by luminescence color changes. Through elegantly choosing metalated organic building blocks, HOFs with permanent porosity can be realized, which largely enriches the structures and functionalities of the HOF library.

Recently, the computational approach has also been employed to screen and predict the structure of HOFs. In principle, the probability of obtaining a structure is directly related to its lattice energy. The lower the energy, the higher the probability that a stacking arrangement can be isolated experimentally. For a given tecton, the possible stacking arrangements could be computationally sampled, whose lattice energy could also be computationally calculated. In this regard, the packing arrangements with porous structures could be discovered by the comparison of the lattice energy of simulated structures. Based on this methodology, Cooper, Day, and their coauthors combined the computational crystal structure prediction (CSP) with a high-throughput crystallization screen to discover HOF polymorphs with high porosity and relatively high stability. Taking two widely used molecules benzene-1,3,5-tricarboxylic acid (TMA) and adamantane-1,3,5,7-tetracarboxylic acid (ADTA) as an example, they successfully predicted and isolated a novel porous polymorph of TMA (denoted as δ-TMA) and three new solvent-inclusion polymorphs of ADTA, verifying the feasibility of this methodology.^34^ Day et al. further combined CSP and property prediction to build energy–structure–function (ESF) maps, which were applied to predict the possible structures and properties of a candidate tecton.^35^ Based on this strategy, three new porous polymorphs of triptycene imidazolone T2 (i.e., T2-β, T2-γ, and T2-δ) and an ultra-low-density form of T2E (i.e., T2E-α) were discovered and experimentally characterized. Especially, T2-γ has a BET surface area of 3425 m^2^ g^–1^, which represents the highest surface area for the reported HOFs. Interestingly, the calculated structures and properties (i.e., nitrogen adsorption isotherms) are in good agreement with those of experimental results. Later, they popularized this strategy to a series of rigid molecules that comprise either a triptycene or a spiro-biphenyl core and are separately functionalized with six different hydrogen-bonding moieties.^36^ Very recently, CSP was further employed to predict and construct a cage-based and low-density (0.54 g cm^–3^) mesoporous HOF (i.e., Cage-3-NH_2_), which has a BET surface area of 1750 m^3^ g^–1^ and represents the first non-interpenetrating mesoporous 3D HOF with permanent porosity.^37^ ESP maps have been shown as a very promising method for discovering novel HOFs, but the cost of acquiring an ESF map is still too high for routine integration into high-throughput virtual screening workflows. To address this problem, Pyzer-Knapp et al. utilized parallel Bayesian optimization to selectively acquire energy and property data, generating the same levels of insight at a fraction of the computational cost.^38^ This approach could be used to improve the navigation of ESP maps and accelerate computational discovery of porous HOFs.

## 3 Stability Enhancement

**Figure 4:**
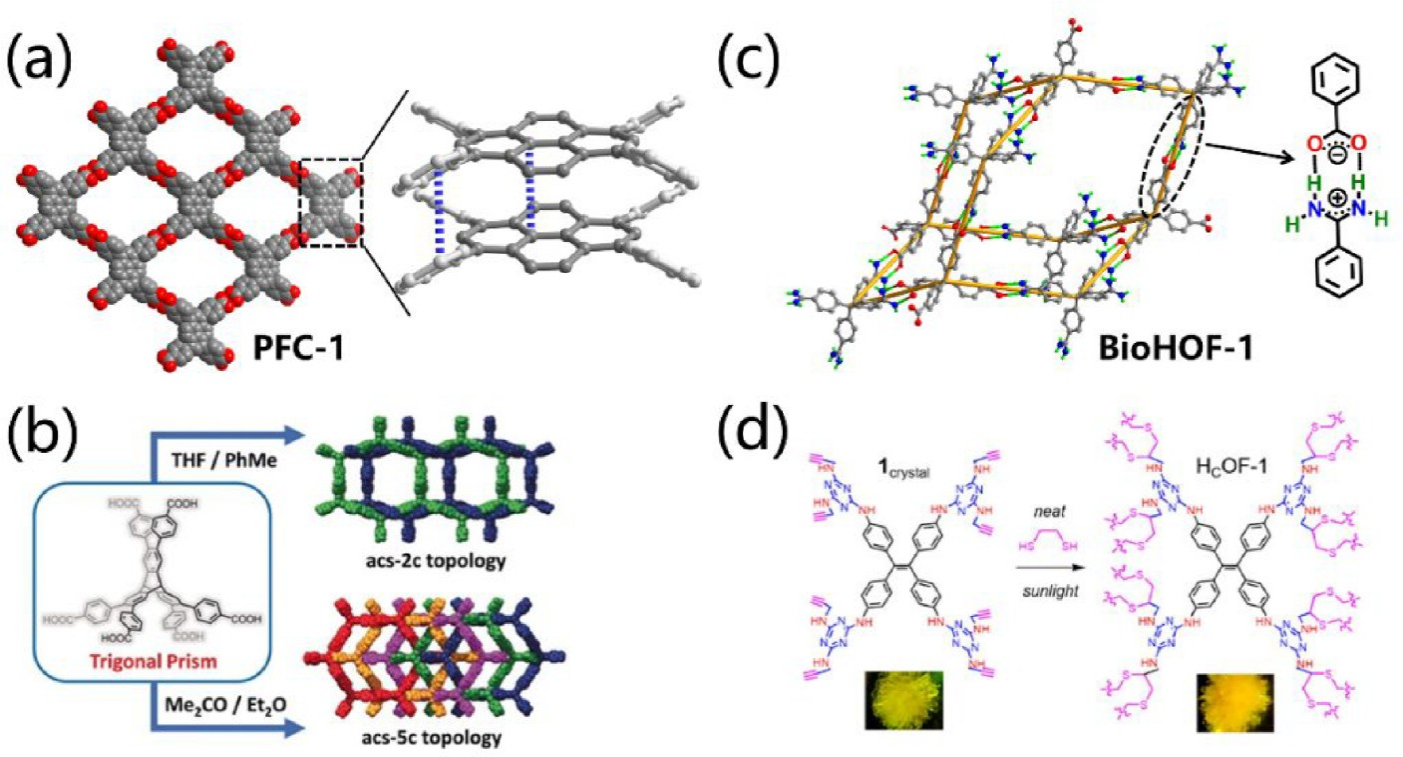
Strategies for enhanced stability of HOFs by the introduction of (a) π–π stacking interaction, (b) multiple interpenetration, (c) electrostatic attraction, and (d) covalent bonding interaction. Panels b and d are reproduced with permission from refs (55) and (56), respectively. Copyright 2019 Wiley-VCH and 2017 American Chemical Society.

Owing to the fragility of linkages, it is difficult for most HOFs to retain their porosity after the removal of guest solvents. Given that low stability is mainly due to weak hydrogen bonds, increasing the numbers of intermolecular hydrogen bonds would unambiguously enhance framework stability. Therefore, using rigid tectons and robust supramolecular synthons with multiple hydrogen pairs is one of the primary strategies to obtain stable HOFs. Besides, the synergy of H-bonds with other intermolecular or even covalent bonding interactions can also largely strengthen the skeleton of HOFs. Several strategies, such as π–π interaction, framework interpenetration, electrostatic interactions, and covalent bonding interactions, have been proposed and utilized to construct porous HOFs (Figure 4). It should be noted that in most cases more than one strategy was employed to synergistically enhance the stability of HOFs. For instance, the π–π interaction, framework interpenetration, and electrostatic interactions were simultaneously introduced into BioHOF-1, making it very stable in aqueous solutions even in boiling water (Figure 4c).^39^ In this part, we will briefly discuss these strategies one by one.

### 3.1 π–π Interactions

Generally, π–π interaction is an attractive interaction between aromatic rings. Therefore, the synergy of H-bonding and π–π stacking interactions is a reliable method to stabilize HOFs. This strategy is particularly applicable when 2D hydrogen-bonded layers are packed together through face to face π–π stacking. In this case, organic tectons are first connected by H-bonds to form 2D hydrogen-bonded layers, which were further packed by π–π stacking interactions to obtain 3D frameworks with 1D open channels along the packing direction. The planar and large π-conjugated tectons with *C*_2_-, *C*_3_-, *C*_4_-, and *C*_6_-symmetry are very suitable for constructing stable HOFs due to their facile accessibility to 2D hydrogen-bonded layers.

The 2D square lattice (*sql* net) is the most common platform used for the construction of stable HOFs.^40−42^ For example, Liu and Cao et al. isolated a crystalline PFC-1 based on 1,3,6,8-tetrakis(*p*-benzoic acid)pyrene (H_4_TBAPy) that comprises a large π-conjugated planar scaffold pyrene and four benzoic acid arms (Figure 4a).^43^ In PFC-1, each H_4_TBAPy is connected by four adjacent ones by carboxyl dimers, forming 2D hydrogen-bonded layers with *sql* topology. The *sql* layers were further packed together in an AA packing mode, resulting in a 3D open framework with an extraordinarily high BET surface area of 2122 m^2^ g^–1^. Due to the shape-matching, both the pyrene scaffold and four benzoic acid arms are perfectly packed with adjacent pyrene and benzoic, respectively, in an AA mode. Therefore, PFC-1 is highly stable, and its structure is not changed after treatment in concentrated HCl for at least 117 days. Chen et al. successfully expanded this platform to HOF-14 by replacing benzoic acid arms with naphthoic acid arms.^44^ HOF-14 has the largest pore aperture (3.1 nm) among the reported HOFs, whose structure can be well retained even after treatment in strong alkaline solutions with a pH of 14. Later, Farha and Li et al. further demonstrated how isoreticular chemistry is applied to this platform for the tunability of HOFs’ structures and properties.^45,46^ Very recently, Liu et al. reported three HOFs, namely, PFC-71–73, which are self-assembled by [5,10,15,20-tetrakis(4-carboxyphenyl)porphyrin] (TCPP) or metallization TCPP (M-TCPP).^47^ Interestingly, the metallization materials PFC-72–73 have a higher stability than metal-free PFC-72 because of more effective π–π stacking between *sql* layers in PFC-72–73 than that in PFC-71.

The hexagonal (*hxl*) net is another platform commonly used for the π–π stacking strategy. Hisaki et al. employed various *C*_3_-symmetric π-conjugated tectons that possess three *o*-bis(4-carboxyphenyl)benzene moieties in the periphery of the core to synthesize isostructural HOFs with hexagonal networks. In these HOFs, the hexagonal layers are stacked without interpenetration, providing permanent porosity for various applications.^48−53^ The honeycomb (*hcb*) net is also a 2D network very suitable for the introduction of π–π stacking interaction. For example, Hashim et al. prepared a series of HOFs with *hcb* topology based on *C*_3_-symmetric organic building blocks and cyclic pyrazolyl trimers, which are highly porous and possess large hexagonal pores.^54^

The strategy is also applicable to 3D networks. For example, Yuan et al. built a 3D ultrastable and easily regenerated HOF-TCBP with *dia* topology for light hydrocarbon adsorption and separation;^57^ Chen and Zhang et al. employed a tetrahedral organic tecton to build a highly stable 3D HOF (HOF-20) with ThSi2 topology for aniline sensing.^58^ Li and Chen et al. synthesized a 3D HOF-76 with a *pcu* network for selective ethane/ethylene separation.^59^ The ultrahigh stability of the above-mentioned 3D HOFs is mainly ascribed to the strong face-to-face π–π stacking between adjacent hydrogen-bonded nets.

### 3.2 Framework Interpenetration

Although framework interpenetration will reduce the pore size and pore void of an HOF, it can improve the framework’s stability because of the multiple-interpenetrated framework being thermodynamically favorable compared with its noninterpenetrated counterpart. Therefore, framework interpenetration is also a common strategy to strengthen HOFs. A typical example is demonstrated by Stoddart et al. They successfully controlled the level of interpenetration by tuning crystallization conditions in the self-assembly of a peripherally extended triptycene H_6_PET {4,4′,4″,4‴,4‴′,4‴″-(9,10-dihydro-9,10-[1,2]benzenoanthracene-2,3,6,7,14,15-hexayl)hexabenzoic acid}.^55^ Two *acs* networks, PETHOF-1 and PETHOF-2, with 2- and 5-fold interpenetration, respectively, were isolated. PETHOF-1 and PETHOF-2 show BET surface areas larger than 1100 m^2^ g^–1^ (Figure 4b). Similarly, Liu and coauthors reported that the self-assembly of H_3_BTB can obtain undulated 2D layers with *hcb* topology, which finally generates PFC-11–13 with different degrees of polycations. Despite the high degree of catenation, PFC-11 and PFC-12 still have BET surface areas of 751.3 and 653.6 m^2^ g^–1^, respectively.^13^ Likewise, Chen et al. reported a 10-fold interpenetrated diamond HOF-30 for the selective adsorption of propyne over propylene, which shows a reversible H-bond and H-bond transformation during the solvent adsorption and desorption processes.^60^

### 3.3 Electrostatic Attractions

The introduction of electrostatic attractions between positive and negative ions is also a viable strategy to enhance the stability of HOFs. Typically, the so-called charge-assisted H-bonding interactions, formed through strong acidic and basic components, is a representative example to introduce electrostatic attractions for strength enhancement of linkages (Figure 4c). So far, several supramolecular synthons containing charge-assisted H-bonding interactions, such as amidinium-carboxylate,^61−64^ guanidium-sulfonate,^65−67^ ammonium-sulfonate,^68^ and amidinium-sulfonate,^69^ have been widely explored to synthesize robust functional HOFs.

### 3.4 Covalent Bonding Interactions

The introduction of covalent bonds to connect tectons in HOFs is also used to improve HOFs’ stability. This strategy was well elucidated by Ke et al. They successfully developed a series of hydrogen-bonded cross-linked organic frameworks (HcOFs) by covalent photoinduced cross-linking organic building blocks that have been preorganized into HOFs (Figure 4d).^56,70^ The resulting HcOFs show a guest-induced elastic expansion and contraction, expanding their voids to adsorb iodine and reversibly recovering their crystalline form after iodine release. Such a unique guest sorption-induced elastic property is also observed in HcOF-6, which originated from the reversible disruption and restoration of the anion clusters upon guest adsorption and desorption.^71^

## 4 Diverse Applications

### 4.1 Gas Storage and Separation

Because the low density of a material favors its high gravimetric storage capacity, the lack of metal ions in HOFs makes them promising adsorbents for gas storage. So far, HOFs have been used to capture various gases including green gas CO_2_,^8,13,28,66,72−76^ clean energy gases like H_2_ and CH_4_,^35,77^ valuable industrial gaseous feedstocks like light hydrocarbon,^78−80^ toxic gases like SO_2_,^81^ etc. SOF-1 is an early attempt of applying HOFs for gas storage, which shows a moderate uptake of CO_2_ (69 cm^3^ g^–1^, STP), C_2_H_2_ (124 cm^3^ g^–1^, STP), and CH_4_ (124 cm^3^ g^–1^, STP) at 1 bar and 195 K.^8^ To improve the gas uptake capacity, a very efficient strategy is increasing the specific surface areas. TTBI,^35^ also denoted as T2-α by Pulido,^77^ with a BET surface area of 2796 m^2^ g^–1^, has a significant adsorption of CO_2_ (81 cm^3^ g^–1^ or 15.9 wt %) at 273 K and 1 bar, as well as H_2_ (10.8 cm^3^ g^–1^ or 2.2 wt %) at 77 K and 1 bar. T2-γ, a polymorph of T2-α, whose BET surface area reaches to 3425 m^2^ g^–1^, has a saturation CH_4_ capacity of 47.4 mol kg^–1^ (437.4 v/v) at 115 K.^77^ KUF-1 can adsorb NH_3_ in a unique type IV fashion at 298 K, which is the first observation in NH_3_ uptake.^65^ Compared with type I sorption behavior found in other materials for NH_3_ sorption, such a unique sorption behavior of KUF-1 has a very high working capacity and recyclability at room temperature.

**Figure 5:**
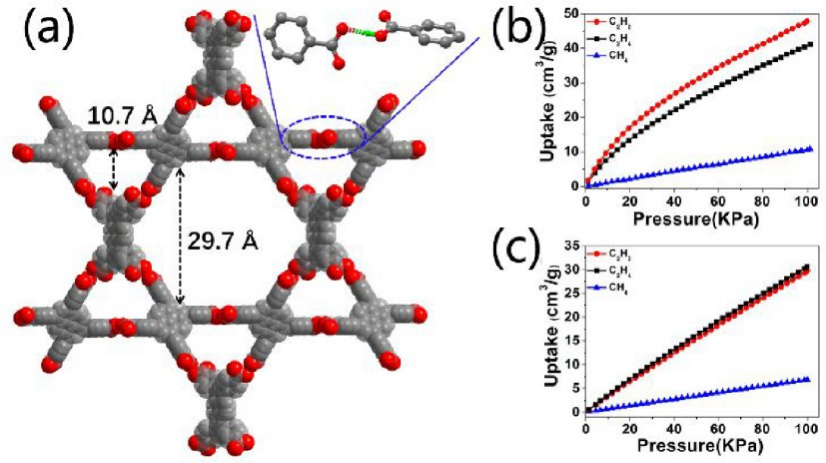
(a) Large channels in PFC-2. C_2_H_2_, C_2_H_4_, and CH_4_ sorption isotherms over (b) PFC-1 and (c) PFC-2, respectively. Reproduced with permission from ref (86). Copyright 2019 American Chemical Society.

Adsorptive separation by porous materials is regarded as one of the most promising separation technologies for its simplicity and energy-saving property. In view of the great importance of light hydrocarbon separation (e.g., C_2_H_4_ and C_3_H_6_ purification), HOFs have been widely utilized for the separation of light hydrocarbons.^29,30,82−85^ When the pore size of HOFs is much larger than light hydrocarbon molecules, the adsorption selectivity usually relies on the preferred binding affinity of host HOFs to guest molecules. For example, HOF-1 with a pore size of ca. 8.2 Å can adsorb 63.2 cm^3^ g^–1^ of C_2_H_2_ but can only adsorb 8.3 cm^3^ g^–1^ of C_2_H_4_ at 273 K and 1 bar, resulting in a high Henry separation selectivity of 19.3 for C_2_H_2_/C_2_H_4_.^1^ Another typical example was demonstrated by PFC-1/PFC-2, which are self-assembled by H_4_TBAPy and have large pore sizes (Figure 5a, 23 Å for PFC-1 and 29 Å for PFC-2).^86^ However, PFC-2 shows a better adsorption of C2 hydrocarbon to CH_4_ than the PFC-1 counterpart due to the presence of unpaired hydrogen bond acceptor C=O groups in the pore surface of PFC-2 (Figure 5b,c). HOF-30a, a 10-fold interpenetrated *dia* network with a pore size of about 4.2 Å, enables the selective adsorption of C_3_H_4_ over C_3_H_6_ with IAST selectivities reaching 7.7 and 7.6 for 1/99 (v/v) and 1/999 of C_3_H_4_/C_3_H_6_ mixtures, respectively, at 298 K.^87^ Other HOFs including HOF-TCBP also display selective adsorption of light hydrocarbons based on preferred interactions between host framework and guest molecules.^57^

**Figure 6:**
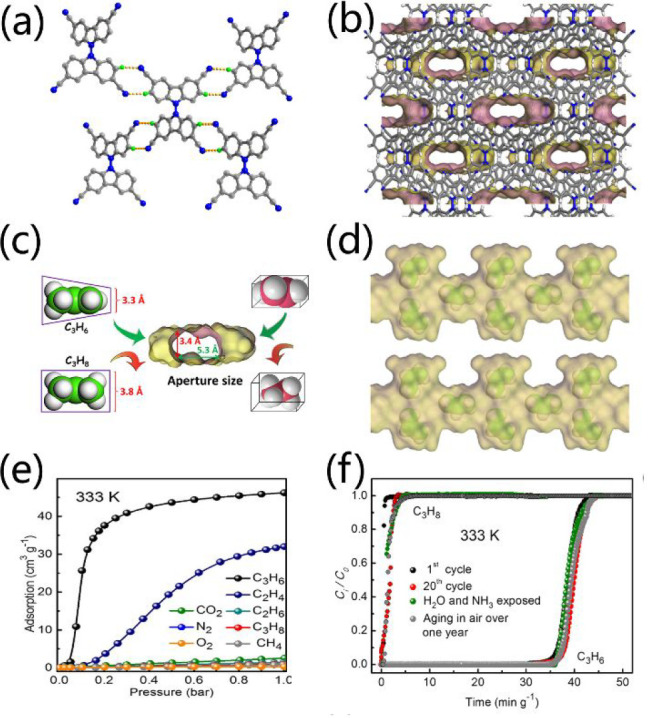
(a) Connection mode of tectons in HOF-FJU-1. (b) 3D architecture of HOF-FJU-1 with 1D narrow channels. (c) Size-dependent separation of C_2_H_4_/C_2_H_6_ and C_3_H_6_/C_3_H_8_ molecules. (d) Adsorbed C_3_H_6_ in HOF-FJU-1. (e) Sorption isotherms of various gases over HOF-FJU-1 at 333 K. (f) Experimental breakthrough results of C_3_H_6_/C_3_H_8_ (50/50, v/v) mixtures. Reproduced with permission from ref (90). Copyright 2022 American Chemical Society.

When the pore size is very small, the sieving effect will largely affect the separation performance of HOFs. In this case, the synergy of the sieving effect and preferential binding affinity could significantly boost the separation performance of HOFs. HOF-21, which exhibits a small pore size of 3.6 Å, can adsorb 1.98 mmol g^–1^ of C_2_H_2_ and 1.27 mmol g^–1^ of C_2_H_4_ at 298 and 1 bar, giving a very high ideal adsorbed solution theory (IAST) C_2_H_2_/C_2_H_4_ selectivity of 7.1.^29^ The outstanding separation performance is mainly ascribed to the sieving effect and the superimposed H-bonding interactions between C_2_H_2_ and HOF-21. Functioning with free −COOH moieties and possessing a small pore size of 6.7 Å, HOF-16 shows a larger C_3_H_6_/C_3_H_8_ uptake difference (by 76%) and higher selectivity (5.4) than the HOF-11 counterpart that was constructed by the same organic tecton but with a larger pore size and without free −COOH decoration.^88^ HOF-FJU-1, constructed by tetracyano bicarbazole, is flexible but very stable even under harsh conditions such as exposure to strong acidity, basicity, and highly polar solvents (Figure 6a).^89^ HOF-FJU-1 displays an evident gate opening behavior at different temperatures. With a suitable pore size of 3.4–3.8 Å (Figure 6b,c), HOF-FJU-1 could solely take up C_2_H_4_ (3.28 Å) while blocking C_2_H_6_ (3.81 Å) in a C_2_H_4_/C_2_H_6_ mixture by the control of operating temperatures and gating pressures (the optimized temperature is 333 K, Figure 6e). Breakthrough experiments show that a high purity of C_2_H_4_ (99.1%) could be obtained at 333 K, making it one of the best porous materials ever reported for C_2_H_4_/C_2_H_6_ separation. HOF-FJU-1 also exhibits highly efficient propylene separation from binary C_3_H_6_/C_3_H_8_ (50/50) with a propylene purity and productivity of over 99.5% and 30.2 L kg^–1^ at 333 K (Figure 6c,d,f), which even can selectively separate C_3_H_6_ and C_2_H_4_ in seven-component CH_4_/C_2_H_4_/C_3_H_6_/C_3_H_6_/C_3_H_8_/CO_2_/H_2_ cracking gas mixtures.^90^ In addition, HOF-FJU-1 can separate C_2_H_2_ from CO_2_ by virtue of their difference in electrostatic potential distribution.^91^ Because of the presence of multiple C–H···π and N···H–C hydrogen-bonded interactions between host HOF-FJU-1 and guest C_2_H_2_, HOF-FJU-1 exhibits extra strong affinity to C_2_H_2_ (46.73 kJ mol^–1^) and has the highest IAST selectivity of 6675 for C_2_H_2_/CO_2_ separation among the reported adsorbents.

**Figure 7:**
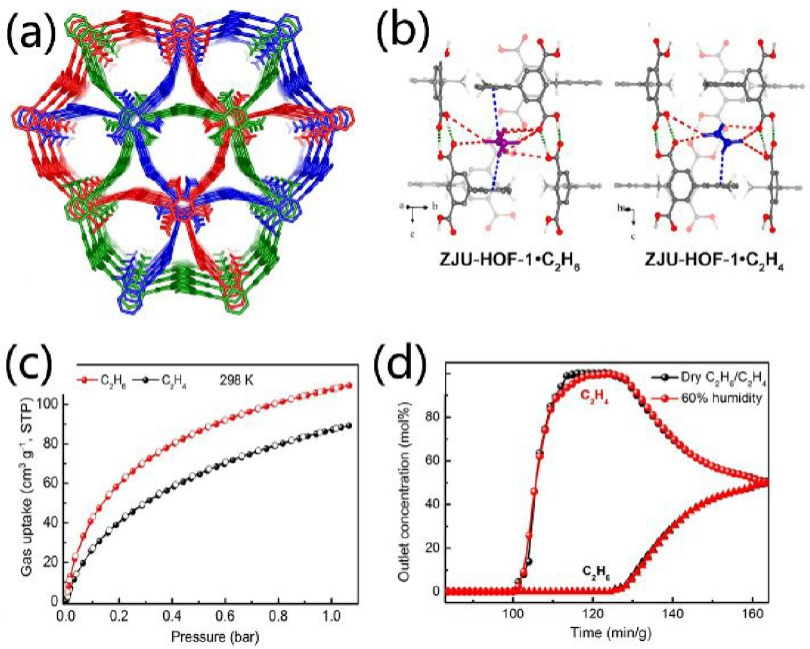
(a) 1D channels in ZJU-HOF-1. (b) Interaction between C_2_H_4_/C_2_H_6_ molecules and host ZJU-HOF-1. (c) Sorption isotherms of C_2_H_4_ and C_2_H_6_ over ZJU-HOF-1 at 298 K, respectively. (d) Experimental breakthrough curves of C_2_H_6_/C_2_H_4_ (50/50, v/v) mixtures at dry conditions and at 60% humidity, respectively. Reproduced with permission from ref (92). Copyright 2021 Wiley-VCH.

Inverse separation like C_2_H_6_-selective or C_3_H_8_-selective adsorption separation is highly desired for C_2_H_6_/C_2_H_4_ and C_3_H_8_/C_3_H_6_ separation because it can simplify the separation process and is energy-saving. Notably, the inverse separation is much more easily realized in HOFs than the MOF counterparts due to the extremely low polarity of the pore surface in HOFs that benefits the selective adsorption of gas with a low dipole or quadrupole moment. So far, several C_2_H_6_-selective or C_3_H_8_-selective HOFs have been successfully developed. For instance, ZJU-HOF-1, which is self-assembled by a hexacarboxylate 2,4,6-trimethylbenzene-1,3,5-triylisophthalate (TMBTI), has 1D triangular channels with a pore size of ca. 7.0 Å and hydrophobic pore surfaces resulting from the decorated methyl groups (Figure 7a).^92^ Interestingly, plentiful cagelike pockets with a diameter of 4.6 Å were found around the channels due to the 3-fold interpenetration and rod-packing configuration. Remarkably, these pockets match better with the larger C_2_H_6_ molecule (4.4 Å) than C_2_H_4_ (4.1 Å), leading to a stronger interaction between host framework and C_2_H_6_ than C_2_H_4_ (Figure 7b). As a consequence of pore confinement and preferential interactions, ZJU-HOF-1 preferentially adsorbs C_2_H_6_ over C_2_H_4_, showing a high C_2_H_6_ uptake of 88 cm^3^ g^–1^ at 0.5 bar and 298 K and a C_2_H_6_/C_2_H_4_ IAST selectivity of 2.25 for C_2_H_6_/C_2_H_4_ (1/1) mixtures at 298 K and 1 bar (Figure 7c). The obtained IAST selectivity is notably higher than those of HOF-BTB (1.4) and HOF-76 (2.0). It is worthy to note that ZJU-HOF-1 can efficiently capture C_2_H_6_ from C_2_H_6_/C_2_H_4_ (1/1) mixtures in ambient conditions under 60% RH, providing a record polymer-grade C_2_H_4_ productivity of 0.98 mmol g^–1^ (Figure 7d). Another example is demonstrated by HOF-76.^59^ HOF-76 can take up 2.95 mmol g^–1^ of C_2_H_6_ but only can adsorb 1.67 mmol g^–1^ of C_2_H_4_, resulting in a high C_2_H_6_/C_2_H_4_ IAST selectivity of 2.0 for 10/90 (v/v) C_2_H_6_/C_2_H_4_ mixtures. Breakthrough experiments show that HOF-76 can directly produce high-purity C_2_H_4_ gas from C_2_H_6_/C_2_H_4_ mixtures with a high productivity of 7.2 L/kg at 298 K and 1.01 bar. Similar behavior was also observed in HIAM-102, where C_2_H_6_ and C_2_H_4_ separation was achieved even under a highly humid condition due to the suitable pore size and the superhydrophobic pore surfaces.^93^

In addition to light hydrocarbon separation, HOFs have also been employed for adsorptive separation of other industrial gases.^94,95^ A flexible and self-healing HOF membrane termed the UPC-HOF-6 membrane was used for H_2_/N_2_ separation.^96^ BTBA-1a and PTBA-1a show highly selective separation of CO_2_/N_2_ with a record high IAST selectivity >2000 under ambient temperature and pressure.^97^ An ultrastable HOF termed TAPM-1 was explored as the stationary phase in the high-solution gas chromatographic separation of benzene and cyclohexane or toluene and methylcyclohexane.^98^ HOF-ZJU-103/HOF-ZJU-104,^31^ HOF-ZJU-201/HOF-ZJU-202,^99^ and HOF-40^100^ exhibit a superior separation performance for Xe/Kr mixtures.

### 4.2 Heterogeneous Catalysis

**Figure 8:**
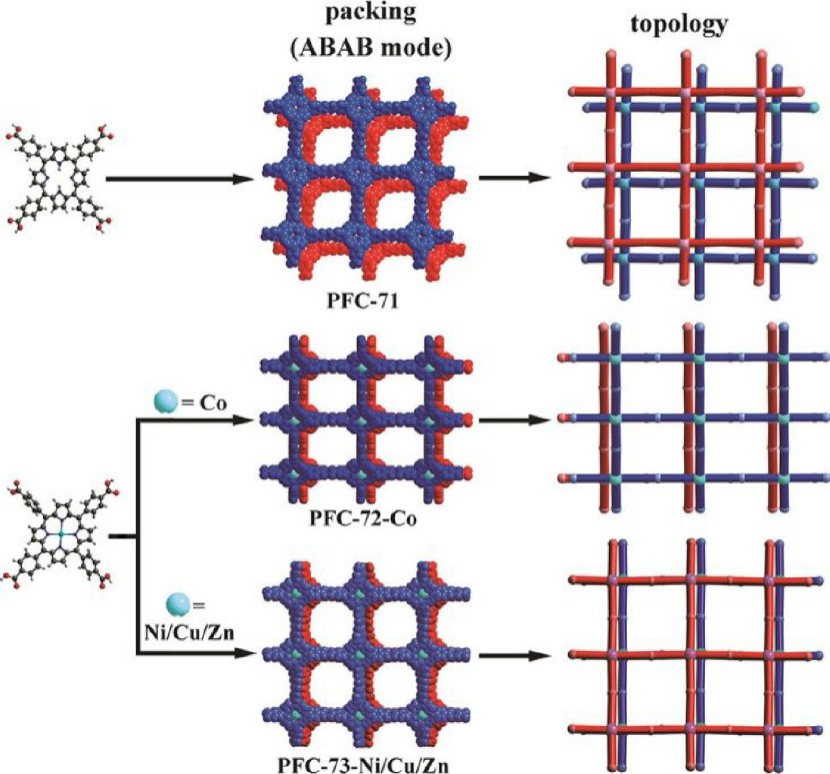
Structures of PFC-71–73. Reproduced with permission from ref (47). Copyright 2022 Wiley-VCH.

**Figure 9:**
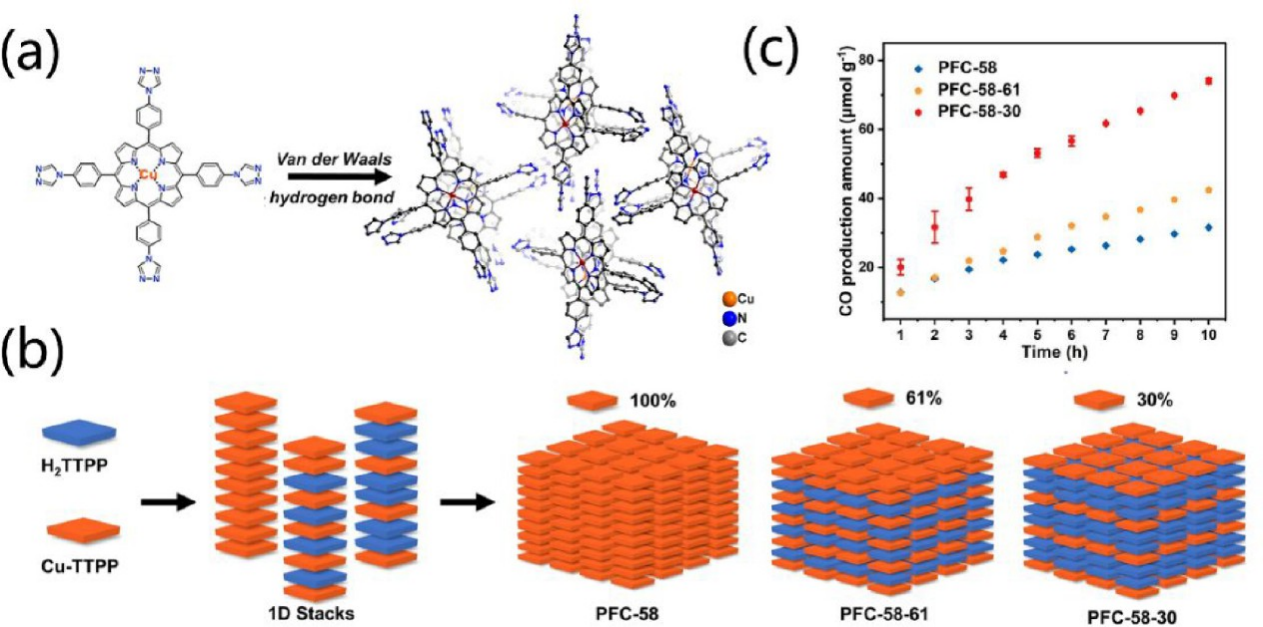
(a) Structure of PFC-58. (b) Tuning the ratio of porphyrin-based organic tecton H_2_TTPP and metalloporphyrin-based tecton Cu-TTPP in PFC-58. (c) Time-dependent CO production over PFC-58, PFC-58–61, and PFC-58–30, respectively. Reproduced with permission from ref (104). Copyright 2022 Wiley-VCH.

The ease of regeneration and recyclability by simple recrystallization as well as solution processability of HOFs enables them to serve as ideal heterogeneous catalysts. One of the primary strategies for preparing HOF-based catalysts is to choose tectons with the intrinsic catalytic activity for the construction of HOFs.^45,101−103^ Porphyrins, a class of heterocyclic molecules with four pyrrole subunits and a large π-conjugated system, can coordinate with various metal ions to form metalloporphyrins, which are of extreme importance in many biological functions due to their light-harvesting, electron transfer, oxygen transport, and various catalytic molecular transformations. Remarkably, the rigid and intrinsic catalytic active features of porphyrins permit their ordered arrangement to form HOFs as heterogeneous catalysts. For instance, a library of highly porous porphyrin HOFs termed PFC-71–73 based on TCPP/M-TCPP have been synthesized in 2022.^47^ In their structures, TCPP or M-TCPP is connected with four other tectons via carboxy dimers to form 2D hydrogen layers with *sql* topology, which further pack together by ABAB mode to obtain 3D open frameworks with pore sizes ranging from 14.9 to 18.8 Å (Figure 8). Metalated PFC-72–73 are highly stable and can retain their structures after being immersed in concentrated HCl, boiling water, or heated to 270 °C. By contrast, the metal-free PFC-71 has a lower stability than PFC-72–73, whose surface area is also much lower than those of metalated PFC-72–73 (600, 1646, and 1714 m^2^ g^–1^ for PFC-71, PFC-72-Co, and PFC-73-Cu, respectively). The observable stability enhancement of PFC-72–73 is mainly ascribed to the more effective π–π stacking among *sql* H-bonded layers in PFC-72–73. As anticipated, PFC-72 and PFC-73 can catalyze the photoreduction of CO_2_ to CO, whose catalytic activity largely depends on the chelated metal species in the porphyrin centers. Later, another stable porphyrin HOF-58 platform that can finely tune the ratio of porphyrin and metalloporphyrin tectons was successfully developed (Figure 9a,b).^104^ The change of metalloporphyrin content in HOF-58 not only alters the microenvironment surrounding of the active sites but also varies the charge separation efficiency. As a result, HOF-58–30 with 30% of metalloporphyrin has the highest activity in the photocatalytic reduction of CO_2_ to HCOOH (29.8 μmol g^–1^ h^–1^) (Figure 9c). Metalloporphyrin-based HOF is also explored as an electrocatalyst. Lan and Chen et al. synthesized a microporous HOF termed Cu-TDPP based on Cu-porphyrin tectons for electrochemical CO_2_ reduction reaction (CO_2_RR) to produce CH_4_.^105^ In the structure of Cu-TDPP, the tectons were connected to each other by two distinct DAT dimers, forming a 2D H-bonded layer with *sql* topology. These layers are further connected together and packed into a 3D framework via interlayer π–π interactions. Notably, a Cu-TDPP nanosheet with a thickness of ca. 5.08 nm can be isolated by the exfoliation of bulk Cu-TDPP. Cu-TDPP nanosheets were successfully applied in electrochemical CO_2_RR, exhibiting a superior FE_CH_4__ of 70% with a high current density (−183.0 mA cm^–2^) at −1.6 V under neutral conditions and maintains FE_CH_4__ > 51% over a wide potential range of −1.5 to −1.7 V. The high performance is mainly ascribed to the numerous H-bonding networks in Cu-TDPP, which benefit proton migration and intermediate stabilization.

Chiral phosphoric acids are widely used as Brønsted acid catalysts for enantioselective reactions. It is of significant interest but still a long-term challenge to load chiral phosphoric acid into porous materials to realize heterogeneous catalysis for the promotion of various challenging reactions. In 2019, Gong et al. synthesized two chiral HOFs, **1**-Ni and **1**-Co, based on chiral phosphoric acid tectons. Strikingly, **1**-Ni and **1**-Co could efficiently catalyze asymmetric [3 + 2] coupling of indoles with quinone monoamine and Friedel–Crafts alkylations of indole with aryl aldimines with 99.9% ee values, respectively.^32^

**Figure 10:**
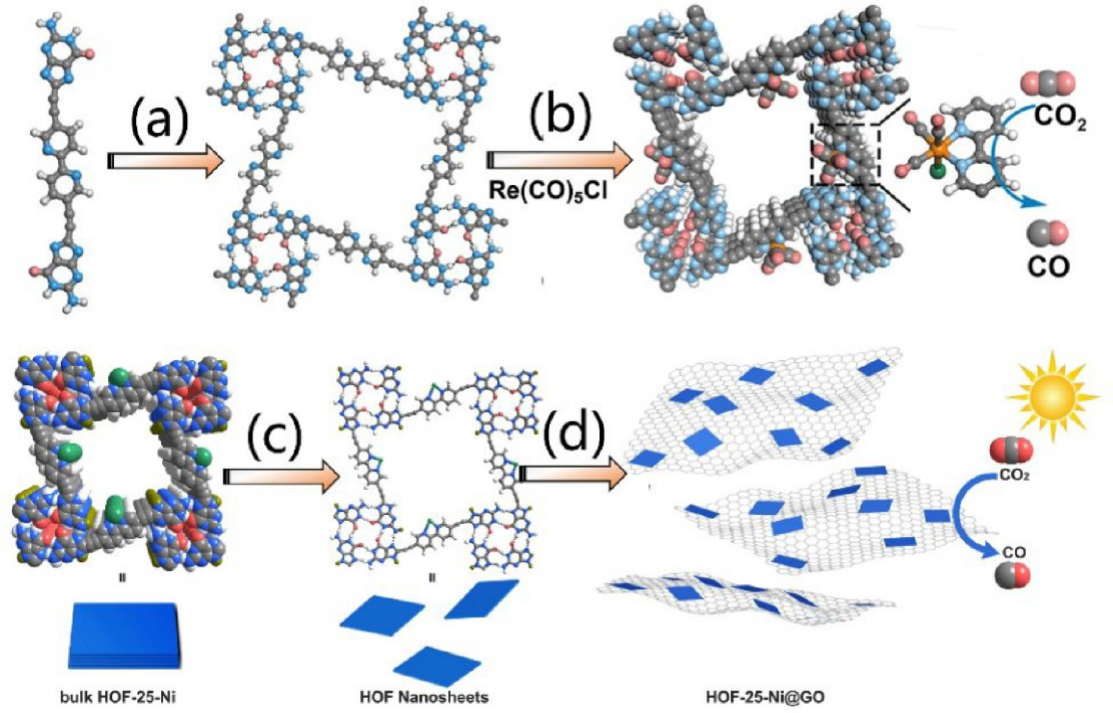
(a) Self-assembly of HOF-25 directed by the H-bonded guanine-quadruplex. (b) Preparation of HOF-25-Re for photoreduction of CO_2_ to CO by a postsynthetic strategy. (c) Exfoliation of HOF-25-Ni into HOF-25-Ni nanosheets by sonication. (d) Dispersing HOF-25-Ni nanosheets to GO for photocatalytic CO_2_ conversion to CO. Reproduced with permission from refs (106 and 107). Copyright 2021 and 2022 Wiley-VCH.

However, the lack of catalytic active sites like metal species in most HOFs significantly impedes their applications in catalysis. In this case, employing stable HOFs as porous solid supports to encapsulate active catalytic species is the other effective strategy to construct HOF-based heterogeneous catalysts. For example, HOF-25, built by 2,2′-bipyridine(bpy)-derived tectons, possesses free 2,2′-bipyridine(bpy) in its pores (Figure 10a).^106^ The tectons are connected with each other by a hydrogen-bonded guanine-quadruplex (G- quadruplex) to form 2D hydrogen-bonded layers with *sql* topology. These layers further pack to each other by AA mode, leading to a 3D open framework with a BET surface of 173 m^2^ g^–1^. Integrating with the guanine-quadruplex and interlayer π–π interaction, HOF-25 is stable and can retain its structure in aqueous solutions with a pH range of 7–11. After reacting with Re(CO)_5_Cl, HOF-25-Re that functioned with photocatalytic active Re(bpy)(CO)_3_Cl moieties is successfully isolated, which can efficiently catalyze CO_2_ photoreduction to CO with both a high CO production rate of 1448 μmol g^–1^ and high selectivity of 93% in the presence of [Ru(bpy)_3_]Cl_2_ under visible light irradiation (Figure 10b). HOF-25 has also been reacted with Ni(II) to get HOF-25-Ni material, in which 20% of bpy was immobilized with Ni(II).^107^ Interestingly, the *sql* layers in HOF-25-Ni can be exfoliated to afford 2D nanosheets (termed HOF-25-Ni NSs) in a high yield of 56% by solvent-assisted sonication method (Figure 10c). The immobilized Ni(II) significantly promote this exfoliation process, which is mainly ascribed to the diminishing interlayer π–π packing interactions by electrostatic repulsion of positive Ni(II) ions. HOF-25-Ni NSs are ultrathin, whose thinness is 4.4 nm. To avoid self-aggregation and facilitate the recycling, HOF-25-Ni NSs were adhered to graphene oxide (GO) with the assistant of strong π–π interactions and Coulomb force (Figure 10d). Strikingly, with the aid of [Ru(bpy)_3_]^2+^ and triisopropanolamine, HOF-25-Ni NSs@GO composite with 10 wt % of NSs can efficiently promote CO_2_ reduction under visible light illumination, showing a 96.3% CO selectivity with a prominent conversion rate up to 24 323 μmol g^–1^ h^–1^. In another example, HOF-19, a cage-based HOF with abundant amino triazine groups, was used to load Pd(II) by postsynthesis.^108^ The resultant Pd(II)@HOF-19 composite exhibits excellent catalytic performance for the Suzuki–Miyaura coupling reactions with isolation yields of 96–98%. The encapsulation of Ru clusters into HOF-19 by postsynthesis obtained Pd@HOF-19, which was used to catalyze hydrogenation of N-heterocyclic compounds including quinolines and indoles.^109^ In addition, the introduction of Pt NPs into PFC-1 by postsynthesis obtained Pt@nano-HOF, which can serve as an efficient photocatalyst for photocatalytic proton reduction.^110,111^ PFC-45/Cu_2_O heterostructure films were also used for efficient CO_2_ photoreduction.^112^

### 4.3 Biomedical Applications

Due to the metal-free nature, most HOFs show low cytotoxicity and high biocompatibility. The reversible H-bonds also enable the gradual degradation of HOFs in a physiological environment. Simultaneously, the porosity of HOFs endows them with the capability to encapsulate diverse guest molecules and controllably release cargos under an external stimulus. In this regard, HOFs are very promising materials for biological applications, such as drug delivery, antibacterial, anticancer, etc.^113,114^

PFC-1 is the first HOF serving as a drug carrier, which was self-assembled by H_4_TBAPy. PFC-1 has a large BET surface area of 2122 m^2^ g^–1^ and 1D rhombic channels with a pore size of 18 × 23 Å^2^, which can efficiently encapsulate doxorubicin (an anticancer drug) with a high uptake of 26.5 wt %. Integrating reactive oxygen species (ROS) generated by the photoactive pyrene skeletons, dox@PFC-1 shows a comparable therapeutic efficacy with the commercial doxorubicin yet considerably lower cytotoxicity because of the synergistic chemo-photodynamic therapy.

**Figure 11:**
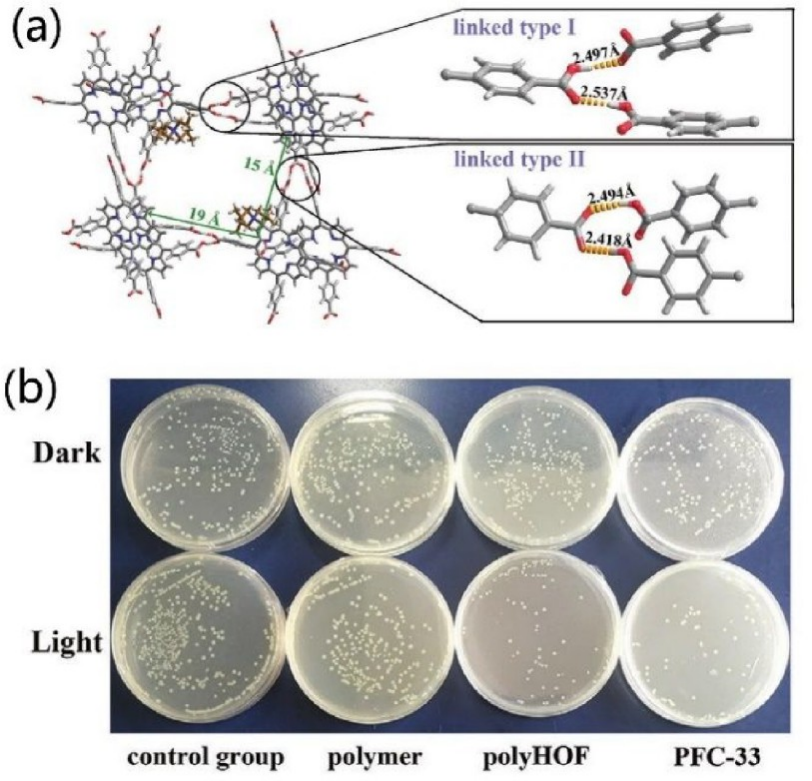
(a) Structure of PFC-33 (the linkages are magnified for clarity). (b) Antibacterial activity of PFC-33 and polyHOF against *E. coli*. under dark or light conditions. Reproduced with permission from ref (115). Copyright 2020 Wiley-VCH.

PFC-33, which is built by H_4_TCPP tecton, exhibits 1D rectangular channels with a pore size of ca. 15 × 19 Å^2^ (Figure 11a). The skeleton of PFC-33 is anionic, and the channels were filled with quaternary ammonium as counterions. Strikingly, due to the presence of porphyrin photosensitizer and quaternary ammonium (a commercial biocide), PFC-33 exhibits synergistic photodynamic and chemical antimicrobial efficiency by virtue of the ROS generation by porphyrin backbone and ion-responsible controlled release of biocide via ion-exchange in various physiological conditions (Figure 11b).^115^ PFC-33 was further fabricated into a polyHOF membrane with the help of the interfacial interaction between the free carboxyl groups on the surface of PFC-33 and the polymer matrix. The resultant flexible but stable polyHOF membrane shows noticeable bacterial inhibition toward *Escherichia coli*.

PFC-55, an HOF constructed by perylenediimide (PDI) tectons, can be persistently maintained in a free radical state and show photothermal and photodynamic capacities under visible light illumination.^116^ To conquer the weak adsorption in the near-infrared (NIR) region, a core–shell heterostructure was fabricated by PFC-55 as shell and an upconversion nanoparticle (i.e., β-NaYF4:Yb,Er) as core via a “bottle-around-ship” strategy, which exhibits a noticeable NIR-responsive photothermal and photodynamic synergistic antimicrobial performance. The photodynamic antibacterial performance is also found in HOF-101-R (R = H, CH_3_, F, NH_2_), which are used for textile coatings.^46^ Interestingly, their antibacterial efficiency could be finely tuned by altering the substituents in tectons.

**Figure 12:**
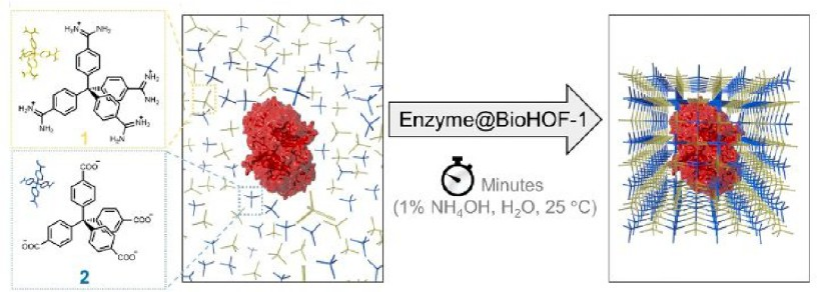
Encapsulation of enzymes into bioHOF-1 through a one-pot synthetic procedure. Reproduced with permission from ref (39). Copyright 2019 American Chemical Society.

HOFs are particularly useful for the protection of or to add new functions of a biomacromolecule via macromolecular encapsulation by virtue of the dynamic interactions and the adaptive guest accommodation ability. Liang et al. first employed HOFs to encapsulate proteins with the aim of stability enhancement.^39^ BioHOF-1 is self-assembled by simply mixing aqueous solutions of tetraamide and tetracarboxylate tectons at ambient temperature, which has pores with an aperture of ca. 6.4 Å (Figure 12). The mild synthetic conditions and the water-soluble tectons of bioHOFs enable it to encapsulate enzymes in aqueous solutions. Various enzymes including catalase (CAT) and alcohol oxidase (AOx) were successfully encapsulated into bioHOF-1 (the loading amount of ca. 6.5% for CAT) through in situ synthesis, which are protected from elevated temperatures, a proteolytic enzyme (trypsin), and a chaotropic agent (urea), yet retain their catalytic activity. Tang et al. reported a nanoscale HOF termed TA-HOFs with a similar structure as another platform to encapsulate various proteins through a similar self-assembly process.^117^ Being encapsulated into the HOF structure, enzymes could retain their original catalytic activities. Moreover, the obtained nanoscale protein@TA-HOFs can efficiently enter into cells, which was exploited for biochemical catalysis in living cells for neuroprotection. Wied et al. adopted N-terminal enzyme fusion with the positively charged module Z_basic2_ to improve the enzyme loading amount in bioHOF-1.^118^ In this seminal work, the pristine d-amino acid oxidase (DAAO) and Z_basic2_ functionalized DAAO (named Z-DAAO) were separately incorporated into bioHOF-1 via the same bottom-up synthetic method. The obtained Z-DAAO@bioHOF-1 shows a 2.5-fold enzyme loading amount (50 wt % of enzyme content) and 6.5-fold specific activity to the DAAO@bioHOF-1 counterpart. Such a strategy was successfully extended to other enzymes, demonstrating the versatility of this strategy in the preparation of biohybrid systems.

**Figure 13:**
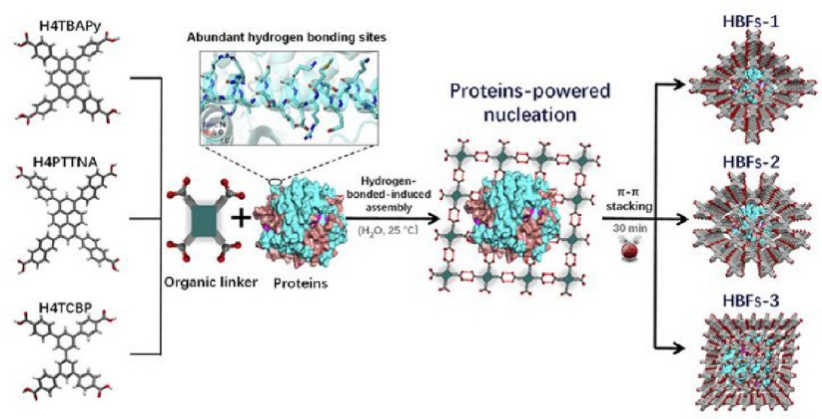
In situ encapsulation of proteins into three carboxyl dimer-based HOFs by a protein-directed assembly strategy. Reproduced with permission from ref (119). Copyright 2021 Elsevier.

Nevertheless, the small pore aperture of the host HOFs and/or relatively low loading amount of proteins still limits the practical application of protein@HOF composites. To tackle these problems, Chen et al. described a versatile protein-directed assembly strategy that enables the encapsulation of proteins into mesoporous HOFs. In this seminal work, various proteins were separately encapsulated into three isostructural mesoporous HOFs to obtain composites termed HBF-1, HBF-2, and HBF-3, respectively (Figure 13).^119^ HBF-1–3 have large accessible pores (e.g., 18.6 Å × 24.5 Å for HBF-1) that inherited from their pristine HOFs, facilitating the mass transportation of various biocatalytic substrates. To demonstrate the universality of this strategy, proteins regardless of shapes and surface chemistry including bovine serum albumin (BSA), cytochrome *c* (Cyt *c*), horseradish peroxidase (HRP), CAT, myoglobin (MB), pepsin, glucose oxidase (GOx), ovalbumin (OVA), and transferrin (TRF) were successfully encapsulated to construct HBFs with a very high protein content ranging from 24.3% to 67.4%. Notably, the encapsulated enzymes show noticeable stability improvement compared to free enzymes. They even show notable advantages for biocatalysis in comparison to those encapsulated in MOFs in terms of ingredient content, robustness, and catalytic efficiency. Such HOF biomimetic entrapment could also modulate the conformation of enzymes (i.e., Cyt *c*), permitting non-native bioactivity for the enzyme.^120^

The protein-directed assembly strategy is also applicable for the simultaneous encapsulation of multiple enzymes into HOFs. For example, Li and Tang et al. adopted the same strategy to encapsulate multiple enzymes into PFC-1 to obtain HOF-confined cascade systems for point-of-care biosensing.^121^ Huang and Yuan et al. employed this strategy to simultaneously encapsulate CAT and GOx into PFC-1 to get EnHOF-101.^122^ After loading the photosensitizer chlorine6 (Ce6), the obtained photo-biocatalytic cascade nanoreactor Ce6@EnHOF-101 exhibits an extraordinary capacity for diabetic would healing through the synergistic PDT.

Besides protein/enzyme encapsulation, neural stem cells (NSCs) have also been successfully encapsulated to an amidinium-carboxylate-based HOF to overcome the low therapeutic efficacy during NSC transplantation.^123^ In this pioneering work, the encapsulated NSCs can be controllably released by NIR-II irradiation. Further experiments on an Alzheimer’s disease mouse model showed that the resultant NSC delivery system could enhance NSC viability, promote neurogenesis, and ameliorate cognitive impairment.

### 4.4 Sensing

**Figure 14:**
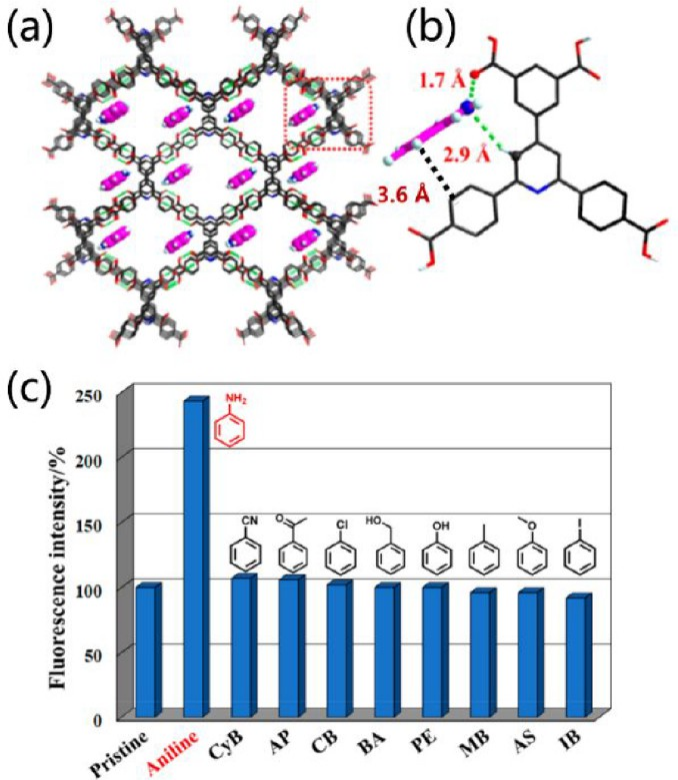
(a) Structure of HOF-20 (the molecules in channels are the adsorbed aniline). (b) Hydrogen bonding and π–π stacking interactions between HOF-20 and the adsorbed aniline. (c) Turn-on sensing mode and sensing selectivity of a proposed sensing system. Reproduced with permission from ref (58). Copyright 2020 American Chemical Society.

Most HOFs are composed of aromatic organic tectons with larger π-conjugated systems. Such building units usually have intrinsic photoluminescent properties, enabling HOFs with the potential as photonic materials.^18,124−128^ It is worthy to note that the luminescent behavior of an HOF could be different from that of its pristine organic build blocks due to the highly ordered arrangement. Specially, HOFs’ luminescence could be responsive to the slight structural change caused by external stimulus or guest–host interactions, which could be harnessed for the luminescent sensing of the stimulus or guest molecules.^129−132^ A representative example is demonstrated by the desolvated CPHATN-1a, which exhibits the reversible acid-induced luminescence change.^49,133^ CPHATN-1 is built from a hexaazatrinaphthylene derivative with carboxyphenyl groups, possessing significant thermal stability up to 633 K. A strong luminescence with a maximum emission at 539 nm is observed for desolvated CPHATN-1, which turns off/on upon the reversible adsorption/desorption of HCl vapor as a result of the protonation/deprotonation of pyradyl N atoms. Another example is HOF-20, which exhibits highly efficient turn-up fluorescent sensing of aniline in aqueous solutions.^58^ Self-assembled by H_4_BCPIA [5-(2,6-bis(4-carboxyphenyl)pyridine-4-yl)isophthalic acid] (Figure 14a), HOF-20 has a BET surface area of 1323 m^2^ g^–1^ and is very stable in water media. Once excited at 315 nm, HOF-20 emits a strong and broad emission centered at ca. 370 nm. Interestingly, the fluorescent intensity of HOF-20 significantly increases after exposure to aniline (Figure 14c). Such “turn-on” sensing is mainly ascribed to the rigidification of the tectons caused by the synergy of the H-bonding and π–π stacking interactions between the aniline and host framework (Figure 14b). In another work, HOF-FAFU-1 was exploited as a fluorescent sensor for hypochlorite detection by a “turn-off” mode.^42^ HOF-FAFU-1, built by 4,4′-OH-TCBP [i.e., 3,3′5,5′-tetrakis(4-carboxyphenyl)-4,4′-dihydroxy-1,1′-biphenyl], is highly stable in aqueous solutions in a pH value ranging from 1 to 9. HOF-FAFU-1 is very sensitive to hypochlorite, whose fluorescent intensities sharply decrease upon the contact with hypochlorite. Such fluorescent quenching is mainly ascribed to the oxidation of 4,4′-OH-TCBP to its nonfluorescent state.

**Figure 15:**
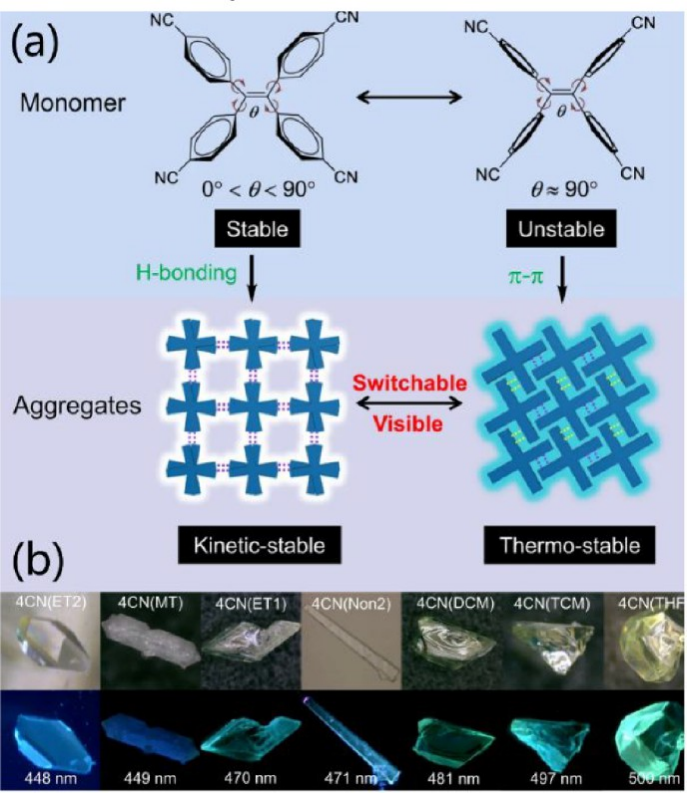
(a) 4CN constructed by a flexible tecton and the tunable emission in switchable HOFs. (b) Photographs of different crystals of 4CN under daylight (first low) and UV light (second low). Reproduced with permission from ref (141). Copyright 2022 Nature Publishing Group.

Tetraphenylethylene (TPE), an aggregation-induced emission (AIE) active molecule, is intensively employed as a backbone to synthesize HOF-based photonic materials with extraordinary luminescence properties.^134−140^ As a flexible molecule, the conformation of TPE is likely to change upon external stimulus by modulating the phenyl rotations as well as the dihedral angles of the ethylene core and phenyl rings. Based on these considerations, Shi et al. have synthesized 11 kinetic-stable and two thermostable HOFs termed 4CN(solvents) with distinct pore structures by a cyano-functionalized TPE organic tecton (Figure 15a).^141^ 4CN(solvents) show a guest-dependent fluorescence due to the different conformations of organic building blocks (Figure 15b). As expected, 4CN is highly sensitive to external stimuli like the exposure to solvent vapors or heating, resulting in the reversible luminescence change (including emission color, lifetime, and brightness) caused by the reversible structural transformations. For instance, 4CN(ET2) will lose ethanol upon heating, associating with the structure rearrangement to 4CN(Non1), and in reverse, the nonporous 4CN(Non1) can also be back to the 4CN(ET2) framework by fuming with dichloromethane/ethanol vapor. Similar behavior is also found in 8PN that was constructed by a nitro-modified TPE organic building blocks.^138^ Another typical example is elucidated by a dynamic 2D woven HOF (another TPE-based HOF termed 2D-90,) which is self-assembled by a tetra-substituted TPE derivative baring phenyl-hydroxy groups.^139^ In this case, 2D-90 exhibits a dark blue fluorescence with a maximum emission at 459 nm upon 365 nm irradiation, which is red-shifted to 474 and 470 nm after the exposure to MeOH and EtOH, respectively. Such guest solvents/vapor regulated photoluminescence is also observed in other TPE-based or non-TPE-derivative dynamic HOFs.^142−144^

**Figure 16:**
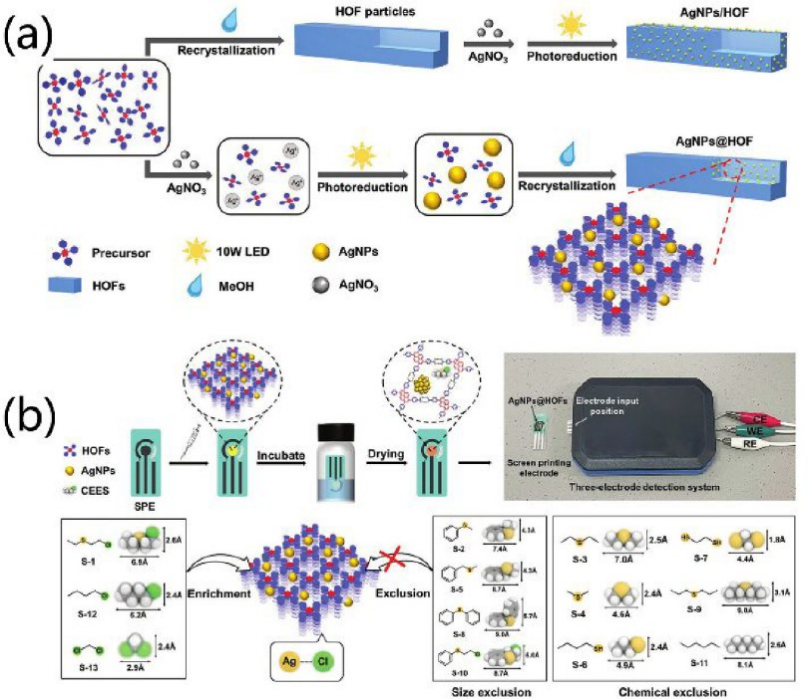
(a) Synthetic process of AgNPs/HOF (top) and AgNPs@HOF (bottom). (b) Fabrication of a portable PEC device based on AgNPs@HOF-101 (top) and the size-exclusion and specific recognition of CEES by the above device (bottom). Reproduced with permission from ref (145). Copyright 2022 Wiley-VCH.

Besides luminescent sensing, the photo- and/or electroactive properties of HOFs can also be used in other analytic methods. Photoelectrochemical (PEC) detection, which is highly sensitive and excellently reproducible, is regarded as a ground-breaking analytic technology for trace analysis. The PEC sensor highly depends on the photoactive materials, which not only should harvest photos to generate photoelectrons but also should identify the analytes. Wang et al. successfully fabricated AgNPs@HOFs nanocomposites as photoactive materials for PEC sensing.^145^ They developed two methods to encapsulate Ag NPs into HOFs (Figure 16a). In the first method, the organic tecton H_4_TBAPy was thoroughly mixed with Ag(I) ions to form a uniform solution, which was illuminated by a 10 W LED for the photoreduction of Ag(I) ions to Ag NPs. Then, a large amount of methanol was added to obtain nanoscale AgNPs@HOF-101. Alternatively, AgNPs/HOF-101 was obtained after changing the synthetic sequence. Interestingly, the loading amount of Ag NPs can be tuned by the control of the LED illumination time, which further affects the photoactivity of the composites. The photocurrent of AgNPs@HOF-101 composites (0.85–1.91 μA) is significantly higher than those of common MOFs including ZIF-8, HKUST-1, MOF-808, and NU-1000 as well as metal oxides including Cu_2_O and ZnO. Notably, the photocurrent is more than 3-fold increased after the Ag NPs encapsulation. AgNPs@HOF-101 was used as photoactive material for PEC sensing (Figure 16b). Strikingly, the portable PEC device based on AgNPs@HOF-101 can selectively recognize 13 different mustard gas simulants on the basis of synergistic size-exclusion and specific recognition. The optimized sensor shows a very low detection limit [15.8 nmol L^–1^ for 2-chloroethyl ethyl sulfide (CEES)], reusability (30 cycles), and long-term working stability (30 days).

Jiang and Wang et al. have synthesized an ultrastable porphyrin-based HOF termed UPC-H4 for selective NO_2_ sensing.^146^ UPC-H4 is constructed by 5,10,15,20-tetrakis(4-(2,4-diaminotriazinyl)phenyl)porphyrin (H_2_TDPP). The desolvated material UPC-H4a can act as an *n*-type semiconductor, whose conductivity highly relies on the concentration of NO_2_. Interestingly, the value of conductivity decrement is linearly related with the NO_2_ content. Therefore, UPC-H4a can serve as an effective sensor for NO_2_ sensing, as demonstrated by a very small LOD (lower than 40 ppb), fast response (17.6 s), and good reproducibility. Remarkably, UPC-H4a exhibits a lower LOD and higher selectivity in the NO_2_ sensing than H_2_TDPP tectons, which is mainly ascribed to the large number of accessible amino sites to interact with NO_2_ in UPC-H4a due to the highly ordered structure.

### 4.5 Proton Conduction

The proton-exchange-membrane fuel cell (PEMFC) is regarded as one of the most promising clean energy resources, in which electrolytes with high proton conductivity and durability are necessary. A primary prerequisite for a material’s proton conduction is the formation of hydrogen-bonded networks that enable the proton movement. In this regard, HOFs naturally become candidates as extraordinary proton conductors due to their inherent hydrogen-bonded networks.^19^ Theoretically, the diverseness of tectons and linkages permits the creation of well-defined H-bonded networks in the channels of HOFs for proton movement, which usually composes functional groups from the host framework and guest molecules filled in pores. So far, HOFs have become an excellent platform for proton conductors, as validated by the successful development of a library of HOFs with high proton productivity and high durability.^147−153^

**Figure 17:**
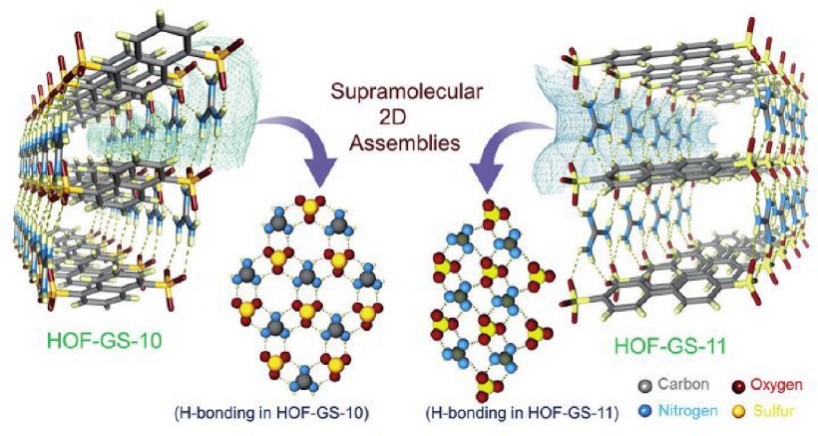
Structure of HOF-GS-10 and HOF-GS-11. Reproduced with permission from ref (154). Copyright 2016 Wiley-VCH.

An appropriate strategy to achieve high proton conductivity for HOFs is optimizing their pore surface’s hydrophobicity to control the amount of water molecules involved in the proton conduction pathways. In an ingenious work, two HOFs, namely, HOF-GS-10 and HOF-GS-11 composed of aryl disulfonate moieties and guanidinium ions, were reported exhibiting ultrahigh proton conduction values of 0.75 × 10^–2^ and 1.8 × 10^–2^ S cm^–1^ at 95% and 30 °C, respectively.^154^ In these two HOFs, the connection of guanidinium cations and the sulfonate moieties by [(G)N–H···O(S)] forms 2D hydrogen-bonded architectures, which further connect to each other by hydrophobic aryl groups that serve as pillars (Figure 17). As a result, 1D channels filled with polar guest molecules can be observed along the crystallographic *a*-axis. Experimental results show that the conductivity of HOF-GS-10 is slightly lower than that of HOF-GS-11 because of the higher hydrophobicity of the naphthalene pillar in HOF-GS-10 than that of the biphenyl pillar in HOF-GS-11. Similar hydrophobicity-dependent proton conductivity is also observed in the other four HOFs assembled by sulfonate anions and ammonium cations.^68^

**Figure 18:**
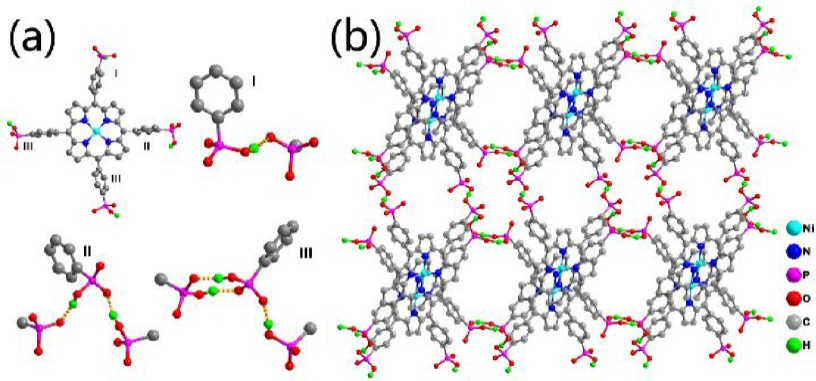
(a) Organic tectons of NiH_8_TPPP and its connection modes. (b) Structure of UPC-H5 (the guest molecules in channels are omitted for clarity).

Another strategy to realize high proton conductivity is changing the guest molecules to optimize the H-bonding networks for proton movement. For instance, porous UPC-H5 based on a nickel tetraphosphonate porphyrin (NiH_8_TPPP) was reported, whose production conductivity is regulated by guest molecules in the channels (Figure 18).^155^ In the structure of UPC-H5, each NiH_4_TPPP^4–^ provides four H atoms from four −PO_3_H moieties and accepts four H atoms from another four −PO_3_H to form eight O–H···O H-bonds, leading to the formation of 2D H-bonded layers. These layers are further connected to adjacent ones to obtain 3D networks by the H-bonding, electrostatic interactions among layers, Me_2_NH^2+^ and lattice water molecules, as well as π–π interactions. The guest DMF and lattice water molecules can be removed to get desolvated UPC-H5a, which further turns to UPC-H5a@NH_3_·H_2_O after exposure to 25% aqueous ammonia. Remarkably, UPC-H5, UPC-H5a, and UPC-H5a@NH_3_·H_2_O have proton conductivities of 1.85 × 10^–3^, 3.42 × 10^–2^, and 1.59 × 10^–1^ S cm^–1^ at 80 °C and 99% RH, respectively.

### 4.6 Other Applications

The merits of HOFs also enable them as multifunctional materials for many other applications, such as electrochromism,^84,156,157^ photochromism,^158^ environmental applications,^9,103,159^ chiral separation,^160^ structural determination,^161^ molecular actuator, etc.^162^ Only several representative examples recently reported are shown below since some of them were summarized by ours’ and others’ review articles.^23,163^

**Figure 19:**
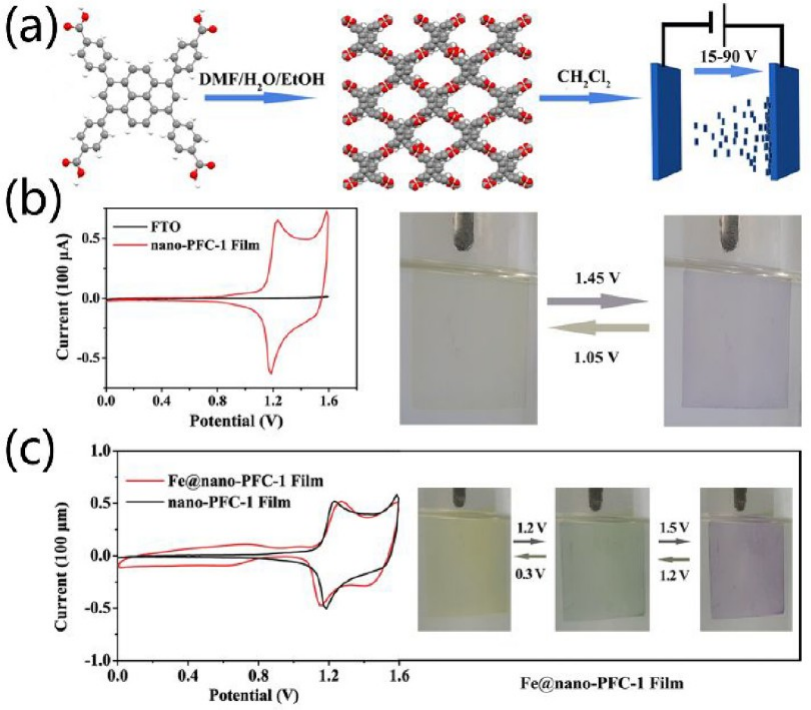
(a) Fabricated process of nano-PFC-1 film by the EPD method. (b) Reversible electrochromic behavior of nano-PFC-1 film. (c) Reversible and successive color change of Fe(II)-modified nano-PFC-1 film under CV scans. Reproduced with permission from ref (157). Copyright 2020 Wiley-VCH.

Electrochromism involves the reversible optical properties produced electrochemically. In a typical electrochromic process, the electroactive species often undergo reversible reduction or oxidation. HOFs are a potential type of electrochromic material when using tectons with intrinsic electrochromic activity. A good example is demonstrated by Feng et al. They fabricated a nano-PFC-1 film that exhibits reversible electrochromic properties by a facile and inexpensive electrophoretic deposition (EPD) method (Figure 19).^156^ The nanoscale PFC-1 materials was first synthesized by a rapid self-assembly of H_4_TBAPy tecton in a mixture of DMF/EtOH/H_2_O, which was used as a raw precursor for electrophoretic deposition (Figure 19a). The negative charge of PFC-1, which derives from internal defects or the deprotonation of carboxylate acid, allows the fabrication of PFC-1 film by the EPD method. As anticipated, nano-PFC-1 film was facilely fabricated by the deposition of nano-PFC-1 on transparent FTO substrates in CH_2_Cl_2_ solutions in 2 min. Strikingly, the obtained nano-PFC-1 film shows a reversible color change from yellow to blue-violet during CV scans (Figure 19b). Moreover, the successive color changes from yellow to green and then to blue-violet were achieved after modification of nanoPFC-1 with Fe(II) (Figure 19c). Besides, the nano-PFC-1 film can be recycled and regenerated by simply rinsing with DMF or DMSO.

**Figure 20:**
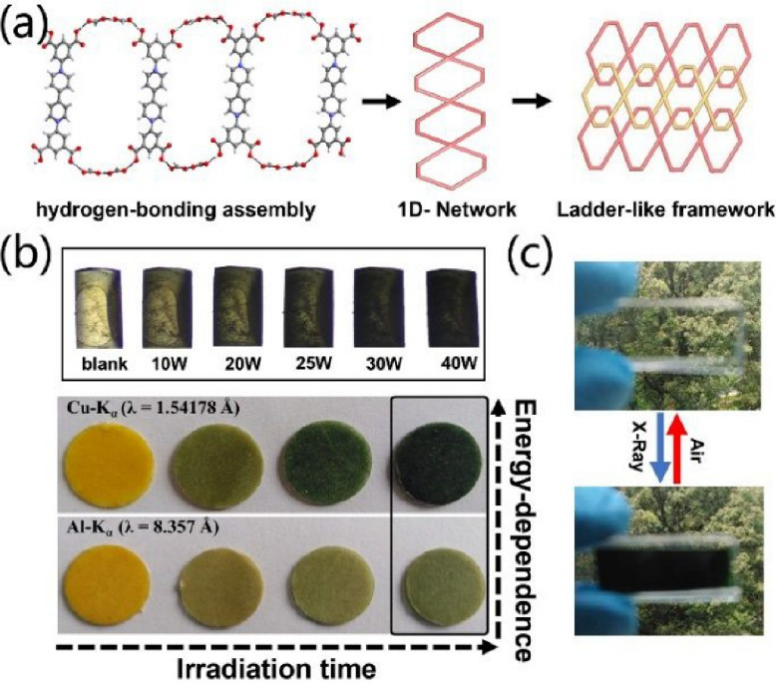
(a) Structure of PFC-25. (b) Color change of PFC-25 under X-ray irradiation with different powers (top) and different wavenumbers (bottom). (c) Observation of X-ray by the naked-eye based on a PFC-25 portable sensor. Reproduced with permission from ref (158). Copyright 2021 Wiley-VCH.

When the reversible color change is induced by photons, the phenomenon is often called photochromism, which also accompanies redox reactions during the color change process. HOFs are potential photochromic materials. For instance, self-assembling by a redox-active viologen ligand, PFC-25 was explored as radiochromic materials, which are highly sensitive to X-ray irradiation (Figure 20a).^158^ The single crystal of PFC-25 shows an obvious color change from light yellow to green in 90 s upon X-ray irradiation (Figure 20b, top). In particular, the energy-dependent color change was also observed for PFC-25 (Figure 20b, bottom). Once irradiated by hard X-ray, PFC-25 shows a color change from yellow to dark green. By contrast, a light green was found when PFC-25 was irradiated by a soft X-ray. Most importantly, PFC-25 can be fabricated into a portable naked-eye sensor for X-ray detection based on the significant color change with ultrahigh sensitivity and good stability (Figure 20c).

**Figure 21:**
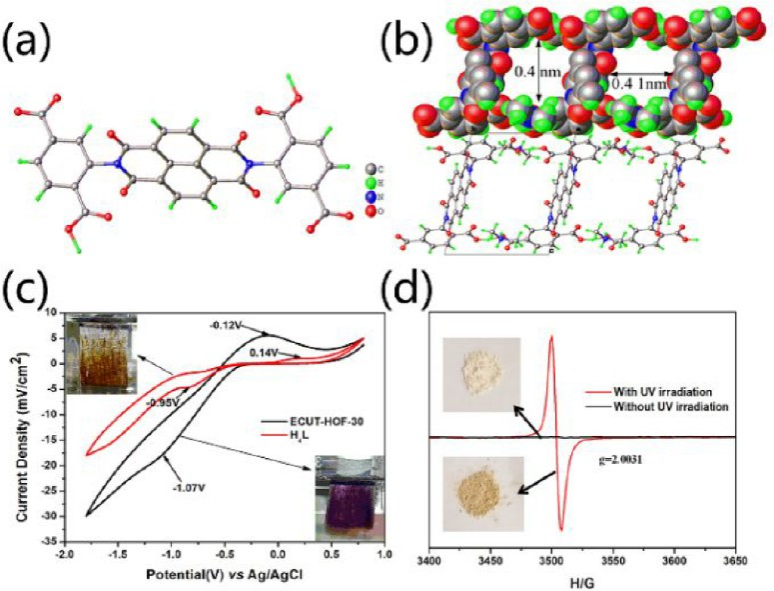
(a) Redox-active tectons adopted in the construction of ECUT-HOF-30. (b) 1D channels in ECUT-HOF-30. Reversible color change for ECUT-HOF-30 under (c) CV scans and (d) UV illumination. Reproduced with permission from ref (84). Copyright 2020 Elsevier.

In some cases, both the electrochromism and photochromism could simultaneously be found in a redox-active HOF. For instance, ECUT-HOF-30, a redox-active HOF, exhibits both photochromic and electrochromic properties (Figure 21).^84^*N*,*N*,-Bis(2-isophthalicacid)naphthalenediimide, an organic tecton decorated with a redox-active unit, was selected to prepare ECUT-HOF-30 (Figure 21a). The obtained ECUT-HOF-30 is microporous and has 1D rectangular channels with a size of ca. 4.0 × 4.1 Å^2^ (Figure 21b). Due to the redox-active unit, ECUT-HOF-30 exhibits an exquisite color change from yellow to purple under CV scans (Figure 21c). Besides, ECUT-HOF-30 is very sensitive to UV light and exhibits a slight color change from light yellow to dark yellow under the UV illumination (365 nm) for 3 min (Figure 21d). During the electrochromic/photochromic process, ECUT-HOF-30 can retain its pristine structure, and the radical formation mainly contributes to the electrochromism and photochromism.

**Figure 22:**
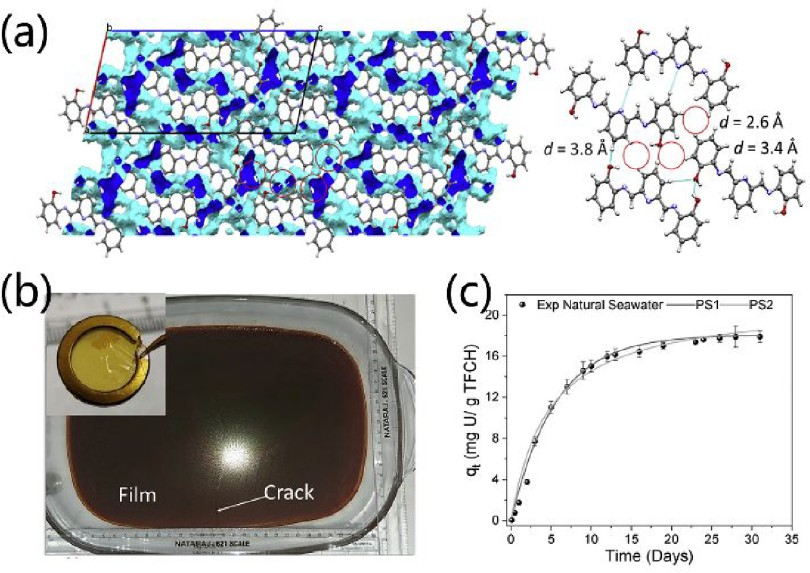
(a) Channels and pockets in ^CSMCRI^HOF-1. (b) Scalable preparation of large-area free-standing thin films of ^CSMCRI^HOF-1 (TFCHs). (c) UES efficiency of TFCHs. Reproduced with permission from ref (165). Copyright 2022 Elsevier.

Uranium extraction from seawater (UES) is still a huge challenge but meaningful, which may effectively alleviate the energy shortage in the future. Due to the ultralow U content (ca. 3.3 ppb), the presence of other competing ions, and high salinity gradient in seawater, the exploration of materials with ultrahigh U affinity and stability is the primary prerequisite to realize UES by a low-cost adsorption process. ^CSMCRI^HOF-1, which is self-assembled by PMAP decorated with pyridine and phenoxy-imine groups, exhibits significant UES capacities (Figure 22).^164^^CSMCRI^HOF-1 is very stable and can retain its structure even under severe acidity, basicity, and saturated salt solutions. A high density of pockets with a diameter of ca. 3.8 Å are present in ^CSMCRI^HOF-1, in which O and N atoms orderly arrange to serve as multidentate sites to bite U (Figure 22a). The presence of inter- and intramolecular hydrogen bonds enables ^CSMCRI^HOF-1 solution processability. Free-standing thin films of ^CSMCRI^HOF-1 (TFCHs) were facilely prepared by solution processing, whose thickness can be tuned from 40 to 500 nm by the control of ^CSMCRI^HOF-1 concentrations and fabricated time (Figure 22b). Surprisingly, the BET surface area also significantly increased from PMAP tecton of 35.8 m^2^ g^–1^ to ^CSMCRI^HOF-1 of 35.8 m^2^ g^–1^ to TFCH of 584.3 m^2^ g^–1^. Thanks to the coordination interactions between N/O and hard U species, the U adsorption over TFCH is mainly ascribed to the chemical adsorption. TFCH exhibits efficient UES capacities of ca. 11 mg g^–1^ with 5 days and 17.9 mg g^–1^ in 30 days from natural seawater (Figure 22c). Remarkably, TFCH can be reused and recycled at least five times with a slight decrease of adsorption efficiency (ca. 11.7%). The above U sorption behaviors make TFCH a new benchmark in UES.

## 5 Conclusion and Outlook

In this Outlook, we have reviewed the use of reticular chemistry and computational methodology to predict and design HOFs. Four main strategies for promoting the stability of HOFs, including the introduction of π–π stacking interaction, electrostatic attraction, covalent bonding interaction, and interpenetration/catenation, have been systematically described. We then discussed the latest advances of HOFs in gas adsorption and separation, heterogeneous catalysis, biological application, sensing, proton conduction, and other applications. Despite great achievements, many problems still need to be addressed. Some challenges and perspectives of HOF development are listed below.

(1) Although reticular chemistry has been successfully used to predict and design HOFs, the precise preparation of HOFs with targeted networks is still a big and long-standing challenge due to the low bonding energy and weakly directional but highly reversible H-bonds. Judiciously choosing tectons with rigid backbone, proper spatial conformation, and synergistic abundant noncovalent interactions is one of the effective solutions. Interestingly, the combination of machine learning and high-throughput crystalline screening may address this problem to some extent and will undoubtedly accelerate the discovery of new functional HOFs. Meanwhile, it is urgent to develop simple but reliable algorithmic models to reduce computational costs and make it practically viable and universal.
(2) There is a great need to discover more porous HOFs that are highly stable to meet requirements of various application scenarios. Although several strategies have been proposed to develop stable HOFs, the number of stable HOFs is still very few in comparison to the large number of stable MOF and COF counterparts. Given that structural stability is critically important to its performance reliability, and is of the primary prerequisites for industrial applications, more effort should be devoted to the discovery of porous HOFs with high stability.
(3) Some tectons bearing intrinsic catalytic active sites like porphyrins have been utilized to build HOFs for heterogeneous catalysis. In many cases, the π–π stacking and framework interpenetration/catenation will keep them from approaching catalytic substrates and therefore detract from the catalytic efficiency. Besides, most HOFs only have small pores, which may limit the mass transportation of substrates in the channels. Moreover, the lack of metal components in most HOFs also largely impedes their applications in heterogeneous catalysis. The encapsulation of catalytically active moieties such as enzymes, noble metal atoms/clusters/nanoparticles, polyoxometalates, and other catalytic active species into stable porous HOFs may address the above problems. Emphasis should be put not only on how to encapsulate the active species into HOFs but also on how these porous matrixes affect their catalytic performances, which may also open a new pathway to explore functional materials.
(4) The stability of HOFs in biological environments needs to be carefully investigated in in vitro and in vivo assays. The long-term biosafety of HOFs also needs to be evaluated for biomedical applications. So far, the size of most reported HOFs is in a millimeter or micrometer scale. Downsizing a bulky HOF to a nanoscale material is very challenging based on current technology. The facile preparation of nanoscale HOFs by either top-down or bottom-up strategies will largely promote their biological and clinic applications.
(5) In consideration of the highly ordered arrangement of tectons, AIE-modified organic building blocks are highly desirable to construct HOF-based photonic materials. In future research, HOFs may show their excellent capability in sensing due to their high structural tunability and precise structure information.

Overall, HOFs provide a powerful platform for an ultra-high-performance material by virtue of their metal-free, highly flexible, solution processable, easily regenerated, and recyclable features. We firmly believe that HOFs will achieve more groundbreaking advancements in the near future.

## References

[ref1] He Y.; Xiang S.; Chen B. A Microporous Hydrogen-Bonded Organic Framework for Highly Selective C_2_H_2_/C_2_H_4_ Separation at Ambient Temperature. J. Am. Chem. Soc. 2011, 133 (37), 14570–14573. 10.1021/ja2066016. 21863860

[ref2] Duchamp D. J.; Marsh R. E. The crystal structure of trimesic acid (benzene-1,3,5-tricarboxylic acid). Acta Crystallogr. B 1969, 25 (1), 5–19. 10.1107/S0567740869001713.

[ref3] Herbstein F. H.; Kapon M.; Reisner G. M. Catenated and non-catenated inclusion complexes of trimesic acid. J. Inclus. Phenom. 1987, 5 (2), 211–214. 10.1007/BF00655650.

[ref4] Ermer O. Five-fold diamond structure of adamantane-1,3,5,7-tetracarboxylic acid. J. Am. Chem. Soc. 1988, 110 (12), 3747–3754. 10.1021/ja00220a005.

[ref5] Simard M.; Su D.; Wuest J. D. Use of hydrogen bonds to control molecular aggregation. Self-assembly of three-dimensional networks with large chambers. J. Am. Chem. Soc. 1991, 113 (12), 4696–4698. 10.1021/ja00012a057.

[ref6] Ermer O.; Lindenberg L. Double-Diamond Inclusion Compounds of 2,6-Dimethylydeneadamantane-1,3,5,7-tetracarboxylic Acid. Helv. Chim. Acta 1991, 74 (4), 825–877. 10.1002/hlca.19910740417.

[ref7] Wuest J. D. Engineering crystals by the strategy of molecular tectonics. Chem. Commun. 2005, (47), 5830–5837. 10.1039/b512641j. 16317453

[ref8] Yang W.; Greenaway A.; Lin X.; Matsuda R.; Blake A. J.; Wilson C.; Lewis W.; Hubberstey P.; Kitagawa S.; Champness N. R.; Schröder M. Exceptional Thermal Stability in a Supramolecular Organic Framework: Porosity and Gas Storage. J. Am. Chem. Soc. 2010, 132 (41), 14457–14469. 10.1021/ja1042935. 20866087

[ref9] Jiang X.-T.; Yin Q.; Liu B.-T.; Chen J.-Y.; Wang R.; Liu T.-F. Porous hydrogen-bonded organic framework membranes for high-performance molecular separation. Nanoscale Adv. 2021, 3 (12), 3441–3446. 10.1039/D1NA00199J. 36133715 PMC9419181

[ref10] Shivakumar K. I.; Noro S.-i.; Yamaguchi Y.; Ishigaki Y.; Saeki A.; Takahashi K.; Nakamura T.; Hisaki I. A hydrogen-bonded organic framework based on redox-active tri(dithiolylidene)cyclohexanetrione. Chem. Commun. 2021, 57 (9), 1157–1160. 10.1039/D0CC07776C. 33411863

[ref11] Slavney A. H.; Kim H. K.; Tao S.; Liu M.; Billinge S. J. L.; Mason J. A. Liquid and Glass Phases of an Alkylguanidinium Sulfonate Hydrogen-Bonded Organic Framework. J. Am. Chem. Soc. 2022, 144 (25), 11064–11068. 10.1021/jacs.2c02918. 35699732

[ref12] Lin R.-B.; Chen B. Hydrogen-bonded organic frameworks: Chemistry and functions. Chem. 2022, 8 (8), 2114–2135. 10.1016/j.chempr.2022.06.015.

[ref13] Li Y.-L.; Alexandrov E. V.; Yin Q.; Li L.; Fang Z.-B.; Yuan W.; Proserpio D. M.; Liu T.-F. Record Complexity in the Polycatenation of Three Porous Hydrogen-Bonded Organic Frameworks with Stepwise Adsorption Behaviors. J. Am. Chem. Soc. 2020, 142 (15), 7218–7224. 10.1021/jacs.0c02406. 32212652

[ref14] Hisaki I.; Xin C.; Takahashi K.; Nakamura T. Designing Hydrogen-Bonded Organic Frameworks (HOFs) with Permanent Porosity. Angew. Chem., Int. Ed. 2019, 58 (33), 11160–11170. 10.1002/anie.201902147. 30891889

[ref15] Lin R.-B.; He Y.; Li P.; Wang H.; Zhou W.; Chen B. Multifunctional porous hydrogen-bonded organic framework materials. Chem. Soc. Rev. 2019, 48 (5), 1362–1389. 10.1039/C8CS00155C. 30676603 PMC11061856

[ref16] Li P.; Ryder M. R.; Stoddart J. F. Hydrogen-Bonded Organic Frameworks: A Rising Class of Porous Molecular Materials. Acc. Mater. Res. 2020, 1 (1), 77–87. 10.1021/accountsmr.0c00019.

[ref17] Wang B.; Lin R.-B.; Zhang Z.; Xiang S.; Chen B. Hydrogen-Bonded Organic Frameworks as a Tunable Platform for Functional Materials. J. Am. Chem. Soc. 2020, 142 (34), 14399–14416. 10.1021/jacs.0c06473. 32786796

[ref18] di Nunzio M. R.; Hisaki I.; Douhal A. HOFs under light: Relevance to photon-based science and applications. J. Photoch. Photobio. C 2021, 47, 100418 10.1016/j.jphotochemrev.2021.100418.

[ref19] Pal S. C.; Mukherjee D.; Sahoo R.; Mondal S.; Das M. C. Proton-Conducting Hydrogen-Bonded Organic Frameworks. ACS Energy Lett. 2021, 6 (12), 4431–4453. 10.1021/acsenergylett.1c02045.

[ref20] Das M. C.; Pal S. C.; Chen B. Emerging microporous HOF materials to address global energy challenges. Joule 2022, 6 (1), 22–27. 10.1016/j.joule.2021.12.005.

[ref21] Gao X.; Lu W.; Wang Y.; Song X.; Wang C.; Kirlikovali K. O.; Li P. Recent advancements in the development of photo- and electro-active hydrogen-bonded organic frameworks. Sci.China Chem. 2022, 65, 2077 10.1007/s11426-022-1333-9.

[ref22] Song X.; Wang Y.; Wang C.; Wang D.; Zhuang G.; Kirlikovali K. O.; Li P.; Farha O. K. Design Rules of Hydrogen-Bonded Organic Frameworks with High Chemical and Thermal Stabilities. J. Am. Chem. Soc. 2022, 144 (24), 10663–10687. 10.1021/jacs.2c02598. 35675383

[ref23] Lin Z.-J.; Cao R. Porous Hydrogen-bonded Organic Frameworks (HOFs): Status and Challenges. Acta Chim. Sinica 2020, 78 (12), 1309–1335. 10.6023/A20080359.

[ref24] Chen Z.; Jiang H.; Li M.; O’Keeffe M.; Eddaoudi M. Reticular Chemistry 3.2: Typical Minimal Edge-Transitive Derived and Related Nets for the Design and Synthesis of Metal-Organic Frameworks. Chem. Rev. 2020, 120 (16), 8039–8065. 10.1021/acs.chemrev.9b00648. 32302477

[ref25] Jiang H.; Alezi D.; Eddaoudi M. A reticular chemistry guide for the design of periodic solids. Nat. Rev. Mater. 2021, 6 (6), 466–487. 10.1038/s41578-021-00287-y.

[ref26] Qin W.-K.; Si D.-H.; Yin Q.; Gao X.-Y.; Huang Q.-Q.; Feng Y.-N.; Xie L.; Zhang S.; Huang X.-S.; Liu T.-F.; Cao R. Reticular Synthesis of Hydrogen-Bonded Organic Frameworks and Their Derivatives via Mechanochemistry. Angew. Chem., Int. Ed. 2022, 61 (27), e202202089 10.1002/anie.202202089. 35460153

[ref27] Thomas-Gipson J.; Beobide G.; Castillo O.; Fröba M.; Hoffmann F.; Luque A.; Pérez-Yáñez S.; Román P. Paddle-Wheel Shaped Copper(II)-Adenine Discrete Entities As Supramolecular Building Blocks To Afford Porous Supramolecular Metal-Organic Frameworks (SMOFs). Cryst. Growth Des. 2014, 14 (8), 4019–4029. 10.1021/cg500634y.

[ref28] Nugent P. S.; Rhodus V. L.; Pham T.; Forrest K.; Wojtas L.; Space B.; Zaworotko M. J. A Robust Molecular Porous Material with High CO_2_ Uptake and Selectivity. J. Am. Chem. Soc. 2013, 135 (30), 10950–10953. 10.1021/ja4054948. 23859072

[ref29] Bao Z.; Xie D.; Chang G.; Wu H.; Li L.; Zhou W.; Wang H.; Zhang Z.; Xing H.; Yang Q.; Zaworotko M. J.; Ren Q.; Chen B. Fine Tuning and Specific Binding Sites with a Porous Hydrogen-Bonded Metal-Complex Framework for Gas Selective Separations. J. Am. Chem. Soc. 2018, 140 (13), 4596–4603. 10.1021/jacs.7b13706. 29540058

[ref30] Liu Y.; Dai J.; Zhang Z.; Yang Y.; Yang Q.; Ren Q.; Bao Z. Crystal Structure Transformation in Hydrogen-bonded Organic Frameworks via Ion Exchange. Chem. Asian J. 2021, 16 (23), 3978–3984. 10.1002/asia.202101151. 34626150

[ref31] Liu Y.; Dai J.; Guo L.; Zhang Z.; Yang Y.; Yang Q.; Ren Q.; Bao Z. Porous Hydrogen-Bonded Frameworks Assembled from Metal-Nucleobase Entities for Xe/Kr Separation. CCS Chemistry 2022, 4 (1), 381–388. 10.31635/ccschem.021.202100824.

[ref32] Gong W.; Chu D.; Jiang H.; Chen X.; Cui Y.; Liu Y. Permanent porous hydrogen-bonded frameworks with two types of Brønsted acid sites for heterogeneous asymmetric catalysis. Nat. Commun. 2019, 10 (1), 600 10.1038/s41467-019-08416-6. 30723208 PMC6363736

[ref33] Yao L.-Y.; Yam V. W.-W. Dual Emissive Gold(I)-Sulfido Cluster Framework Capable of Benzene-Cyclohexane Separation in the Solid State Accompanied by Luminescence Color Changes. J. Am. Chem. Soc. 2021, 143 (6), 2558–2566. 10.1021/jacs.0c11891. 33533605

[ref34] Cui P.; McMahon D. P.; Spackman P. R.; Alston B. M.; Little M. A.; Day G. M.; Cooper A. I. Mining predicted crystal structure landscapes with high throughput crystallisation: old molecules, new insights. Chem. Sci. 2019, 10 (43), 9988–9997. 10.1039/C9SC02832C. 32055355 PMC6991173

[ref35] Pulido A.; Chen L.; Kaczorowski T.; Holden D.; Little M. A.; Chong S. Y.; Slater B. J.; McMahon D. P.; Bonillo B.; Stackhouse C. J.; Stephenson A.; Kane C. M.; Clowes R.; Hasell T.; Cooper A. I.; Day G. M. Functional materials discovery using energy-structure-function maps. Nature 2017, 543 (7647), 657–664. 10.1038/nature21419. 28329756 PMC5458805

[ref36] Zhao C.; Chen L.; Che Y.; Pang Z.; Wu X.; Lu Y.; Liu H.; Day G. M.; Cooper A. I. Digital navigation of energy-structure-function maps for hydrogen-bonded porous molecular crystals. Nat. Commun. 2021, 12 (1), 817 10.1038/s41467-021-21091-w. 33547307 PMC7865007

[ref37] Zhu Q.; Johal J.; Widdowson D. E.; Pang Z.; Li B.; Kane C. M.; Kurlin V.; Day G. M.; Little M. A.; Cooper A. I. Analogy Powered by Prediction and Structural Invariants: Computationally Led Discovery of a Mesoporous Hydrogen-Bonded Organic Cage Crystal. J. Am. Chem. Soc. 2022, 144 (22), 9893–9901. 10.1021/jacs.2c02653. 35634799 PMC9490843

[ref38] Pyzer-Knapp E. O.; Chen L.; Day G. M.; Cooper A. I. Accelerating computational discovery of porous solids through improved navigation of energy-structure-function maps. Sci. Adv. 2021, 7 (33), eabi4763 10.1126/sciadv.abi4763. 34389543 PMC8363149

[ref39] Liang W.; Carraro F.; Solomon M. B.; Bell S. G.; Amenitsch H.; Sumby C. J.; White N. G.; Falcaro P.; Doonan C. J. Enzyme Encapsulation in a Porous Hydrogen-Bonded Organic Framework. J. Am. Chem. Soc. 2019, 141 (36), 14298–14305. 10.1021/jacs.9b06589. 31426638

[ref40] Suzuki Y.; Tohnai N.; Saeki A.; Hisaki I. Hydrogen-bonded organic frameworks of twisted polycyclic aromatic hydrocarbon. Chem. Commun. 2020, 56 (87), 13369–13372. 10.1039/D0CC06081J. 33030481

[ref41] Ji Q.; Takahashi K.; Noro S.-i.; Ishigaki Y.; Kokado K.; Nakamura T.; Hisaki I. A Hydrogen-Bonded Organic Framework Based on Pyrazinopyrazine. Cryst. Growth Des. 2021, 21 (8), 4656–4664. 10.1021/acs.cgd.1c00506.

[ref42] Lin Z.-J.; Qin J.-Y.; Zhan X.-P.; Wu K.; Cao G.-J.; Chen B. Robust Mesoporous Functional Hydrogen-Bonded Organic Framework for Hypochlorite Detection. ACS Appl. Mater. Interfaces 2022, 14 (18), 21098–21105. 10.1021/acsami.2c05176. 35482947

[ref43] Yin Q.; Zhao P.; Sa R.-J.; Chen G.-C.; Lü J.; Liu T.-F.; Cao R. An Ultra-Robust and Crystalline Redeemable Hydrogen-Bonded Organic Framework for Synergistic Chemo-Photodynamic Therapy. Angew. Chem., Int. Ed. 2018, 57 (26), 7691–7696. 10.1002/anie.201800354. 29696754

[ref44] Wang B.; Lv X.-L.; Lv J.; Ma L.; Lin R.-B.; Cui H.; Zhang J.; Zhang Z.; Xiang S.; Chen B. A novel mesoporous hydrogen-bonded organic framework with high porosity and stability. Chem. Commun. 2020, 56 (1), 66–69. 10.1039/C9CC07802A. 31790104

[ref45] Ma K.; Li P.; Xin J. H.; Chen Y.; Chen Z.; Goswami S.; Liu X.; Kato S.; Chen H.; Zhang X.; Bai J.; Wasson M. C.; Maldonado R. R.; Snurr R. Q.; Farha O. K. Ultrastable Mesoporous Hydrogen-Bonded Organic Framework-Based Fiber Composites toward Mustard Gas Detoxification. Cell Rep. Phys. Sci. 2020, 1 (2), 100024 10.1016/j.xcrp.2020.100024.

[ref46] Wang Y.; Ma K.; Bai J.; Xu T.; Han W.; Wang C.; Chen Z.; Kirlikovali K. O.; Li P.; Xiao J.; Farha O. K. Chemically Engineered Porous Molecular Coatings as Reactive Oxygen Species Generators and Reservoirs for Long-Lasting Self-Cleaning Textiles. Angew. Chem., Int. Ed. 2022, 61 (8), e202115956 10.1002/anie.202115956. 34931436

[ref47] Yin Q.; Alexandrov E. V.; Si D.-H.; Huang Q.-Q.; Fang Z.-B.; Zhang Y.; Zhang A.-A.; Qin W.-K.; Li Y.-L.; Liu T.-F.; Proserpio D. M. Metallization-Prompted Robust Porphyrin-Based Hydrogen-Bonded Organic Frameworks for Photocatalytic CO_2_ Reduction. Angew. Chem., Int. Ed. 2022, 61 (6), e202115854 10.1002/anie.202115854. 34877789

[ref48] Suzuki Y.; Gutiérrez M.; Tanaka S.; Gomez E.; Tohnai N.; Yasuda N.; Matubayasi N.; Douhal A.; Hisaki I. Construction of isostructural hydrogen-bonded organic frameworks: limitations and possibilities of pore expansion. Chem. Sci. 2021, 12 (28), 9607–9618. 10.1039/D1SC02690A. 34349933 PMC8293819

[ref49] Hisaki I.; Suzuki Y.; Gomez E.; Ji Q.; Tohnai N.; Nakamura T.; Douhal A. Acid Responsive Hydrogen-Bonded Organic Frameworks. J. Am. Chem. Soc. 2019, 141 (5), 2111–2121. 10.1021/jacs.8b12124. 30615836

[ref50] Hisaki I.; Nakagawa S.; Ikenaka N.; Imamura Y.; Katouda M.; Tashiro M.; Tsuchida H.; Ogoshi T.; Sato H.; Tohnai N.; Miyata M. A Series of Layered Assemblies of Hydrogen-Bonded, Hexagonal Networks of C3-Symmetric π-Conjugated Molecules: A Potential Motif of Porous Organic Materials. J. Am. Chem. Soc. 2016, 138 (20), 6617–6628. 10.1021/jacs.6b02968. 27133443

[ref51] Hisaki I.; Nakagawa S.; Sato H.; Tohnai N. Alignment of paired molecules of C60 within a hexagonal platform networked through hydrogen-bonds. Chem. Commun. 2016, 52 (63), 9781–9784. 10.1039/C6CC04310K. 27417325

[ref52] Hisaki I.; Ikenaka N.; Tsuzuki S.; Tohnai N. Sterically crowded hydrogen-bonded hexagonal network frameworks. Mater. Chem. Front. 2018, 2 (2), 338–346. 10.1039/C7QM00544J.

[ref53] Hisaki I.; Toda H.; Sato H.; Tohnai N.; Sakurai H. A Hydrogen-Bonded Hexagonal Buckybowl Framework. Angew. Chem., Int. Ed. 2017, 56 (48), 15294–15298. 10.1002/anie.201708115. 29024384

[ref54] Hashim M. I.; Le H. T. M.; Chen T.-H.; Chen Y.-S.; Daugulis O.; Hsu C.-W.; Jacobson A. J.; Kaveevivitchai W.; Liang X.; Makarenko T.; Miljanić O. Š.; Popovs I.; Tran H. V.; Wang X.; Wu C.-H.; Wu J. I. Dissecting Porosity in Molecular Crystals: Influence of Geometry, Hydrogen Bonding, and [π···π] Stacking on the Solid-State Packing of Fluorinated Aromatics. J. Am. Chem. Soc. 2018, 140 (18), 6014–6026. 10.1021/jacs.8b02869. 29656637

[ref55] Li P.; Li P.; Ryder M. R.; Liu Z.; Stern C. L.; Farha O. K.; Stoddart J. F. Interpenetration Isomerism in Triptycene-Based Hydrogen-Bonded Organic Frameworks. Angew. Chem., Int. Ed. 2019, 58 (6), 1664–1669. 10.1002/anie.201811263. 30548232

[ref56] Lin Y.; Jiang X.; Kim S. T.; Alahakoon S. B.; Hou X.; Zhang Z.; Thompson C. M.; Smaldone R. A.; Ke C. An Elastic Hydrogen-Bonded Cross-Linked Organic Framework for Effective Iodine Capture in Water. J. Am. Chem. Soc. 2017, 139 (21), 7172–7175. 10.1021/jacs.7b03204. 28506061

[ref57] Hu F.; Liu C.; Wu M.; Pang J.; Jiang F.; Yuan D.; Hong M. An Ultrastable and Easily Regenerated Hydrogen-Bonded Organic Molecular Framework with Permanent Porosity. Angew. Chem., Int. Ed. 2017, 56 (8), 2101–2104. 10.1002/anie.201610901. 28090721

[ref58] Wang B.; He R.; Xie L.-H.; Lin Z.-J.; Zhang X.; Wang J.; Huang H.; Zhang Z.; Schanze K. S.; Zhang J.; Xiang S.; Chen B. Microporous Hydrogen-Bonded Organic Framework for Highly Efficient Turn-Up Fluorescent Sensing of Aniline. J. Am. Chem. Soc. 2020, 142 (28), 12478–12485. 10.1021/jacs.0c05277. 32551570

[ref59] Zhang X.; Li L.; Wang J.-X.; Wen H.-M.; Krishna R.; Wu H.; Zhou W.; Chen Z.-N.; Li B.; Qian G.; Chen B. Selective Ethane/Ethylene Separation in a Robust Microporous Hydrogen-Bonded Organic Framework. J. Am. Chem. Soc. 2020, 142 (1), 633–640. 10.1021/jacs.9b12428. 31838841 PMC11061857

[ref60] Yang W.; Wang J.; Wang H.; Bao Z.; Zhao J. C.-G.; Chen B. Highly Interpenetrated Robust Microporous Hydrogen-Bonded Organic Framework for Gas Separation. Cryst. Growth Des. 2017, 17 (11), 6132–6137. 10.1021/acs.cgd.7b01322.

[ref61] Ma L.; Arman H.; Xie Y.; Zhou W.; Chen B. Solvent-Dependent Self-Assembly of Hydrogen-Bonded Organic Porphyrinic Frameworks. Cryst. Growth Des. 2022, 22, 3808 10.1021/acs.cgd.2c00182.

[ref62] Nicks J.; Boer S. A.; White N. G.; Foster J. A. Monolayer nanosheets formed by liquid exfoliation of charge-assisted hydrogen-bonded frameworks. Chem. Sci. 2021, 12 (9), 3322–3327. 10.1039/D0SC06906J. 34164102 PMC8179369

[ref63] Boer S. A.; Morshedi M.; Tarzia A.; Doonan C. J.; White N. G. Molecular Tectonics: A Node-and-Linker Building Block Approach to a Family of Hydrogen-Bonded Frameworks. Chem.—Eur. J. 2019, 25 (42), 10006–10012. 10.1002/chem.201902117. 31267583

[ref64] Boer S. A.; Wang P.-X.; MacLachlan M. J.; White N. G. Open Pentiptycene Networks Assembled through Charge-Assisted Hydrogen Bonds. Cryst. Growth Des. 2019, 19 (8), 4829–4835. 10.1021/acs.cgd.9b00801.

[ref65] Kang D. W.; Kang M.; Kim H.; Choe J. H.; Kim D. W.; Park J. R.; Lee W. R.; Moon D.; Hong C. S. A Hydrogen-Bonded Organic Framework (HOF) with Type IV NH_3_ Adsorption Behavior. Angew. Chem., Int. Ed. 2019, 58 (45), 16152–16155. 10.1002/anie.201911087. 31502347

[ref66] Brekalo I.; Deliz D. E.; Barbour L. J.; Ward M. D.; Friščić T.; Holman K. T. Microporosity of a Guanidinium Organodisulfonate Hydrogen-Bonded Framework. Angew. Chem., Int. Ed. 2020, 59 (5), 1997–2002. 10.1002/anie.201911861. 31663253

[ref67] Adachi T.; Ward M. D. Versatile and Resilient Hydrogen-Bonded Host Frameworks. Acc. Chem. Res. 2016, 49 (12), 2669–2679. 10.1021/acs.accounts.6b00360. 27689535

[ref68] Xing G.; Yan T.; Das S.; Ben T.; Qiu S. Synthesis of Crystalline Porous Organic Salts with High Proton Conductivity. Angew. Chem., Int. Ed. 2018, 57 (19), 5345–5349. 10.1002/anie.201800423. 29532575

[ref69] Xing G.; Bassanetti I.; Bracco S.; Negroni M.; Bezuidenhout C.; Ben T.; Sozzani P.; Comotti A. A double helix of opposite charges to form channels with unique CO_2_ selectivity and dynamics. Chem. Sci. 2019, 10 (3), 730–736. 10.1039/C8SC04376K. 30809339 PMC6354830

[ref70] Jiang X.; Cui X.; Duncan A. J. E.; Li L.; Hughes R. P.; Staples R. J.; Alexandrov E. V.; Proserpio D. M.; Wu Y.; Ke C. Topochemical Synthesis of Single-Crystalline Hydrogen-Bonded Cross-Linked Organic Frameworks and Their Guest-Induced Elastic Expansion. J. Am. Chem. Soc. 2019, 141 (27), 10915–10923. 10.1021/jacs.9b05232. 31246447

[ref71] Samanta J.; Dorn R. W.; Zhang W.; Jiang X.; Zhang M.; Staples R. J.; Rossini A. J.; Ke C. An ultra-dynamic anion-cluster-based organic framework. Chem. 2022, 8 (1), 253–267. 10.1016/j.chempr.2021.11.014.

[ref72] Luo X.-Z.; Jia X.-J.; Deng J.-H.; Zhong J.-L.; Liu H.-J.; Wang K.-J.; Zhong D.-C. A Microporous Hydrogen-Bonded Organic Framework: Exceptional Stability and Highly Selective Adsorption of Gas and Liquid. J. Am. Chem. Soc. 2013, 135 (32), 11684–11687. 10.1021/ja403002m. 23885835

[ref73] Lü J.; Perez-Krap C.; Suyetin M.; Alsmail N. H.; Yan Y.; Yang S.; Lewis W.; Bichoutskaia E.; Tang C. C.; Blake A. J.; Cao R.; Schröder M. A Robust Binary Supramolecular Organic Framework (SOF) with High CO_2_ Adsorption and Selectivity. J. Am. Chem. Soc. 2014, 136 (37), 12828–12831. 10.1021/ja506577g. 25184689 PMC4183619

[ref74] Nandi S.; Chakraborty D.; Vaidhyanathan R. A permanently porous single molecule H-bonded organic framework for selective CO_2_ capture. Chem. Commun. 2016, 52 (45), 7249–7252. 10.1039/C6CC02964G. 27174692

[ref75] Yang W.; Li B.; Wang H.; Alduhaish O.; Alfooty K.; Zayed M. A.; Li P.; Arman H. D.; Chen B. A Microporous Porphyrin-Based Hydrogen-Bonded Organic Framework for Gas Separation. Cryst. Growth Des. 2015, 15 (4), 2000–2004. 10.1021/acs.cgd.5b00147.

[ref76] Wang H.; Wu H.; Kan J.; Chang G.; Yao Z.; Li B.; Zhou W.; Xiang S.; Cong-Gui Zhao J.; Chen B. A microporous hydrogen-bonded organic framework with amine sites for selective recognition of small molecules. J. Mater. Chem. A 2017, 5 (18), 8292–8296. 10.1039/C7TA01364G.

[ref77] Mastalerz M.; Oppel I. M. Rational Construction of an Extrinsic Porous Molecular Crystal with an Extraordinary High Specific Surface Area. Angew. Chem., Int. Ed. 2012, 51 (21), 5252–5255. 10.1002/anie.201201174. 22473702

[ref78] Liu Y.; Xu Q.; Chen L.; Song C.; Yang Q.; Zhang Z.; Lu D.; Yang Y.; Ren Q.; Bao Z. Hydrogen-bonded metal-nucleobase frameworks for highly selective capture of ethane/propane from methane and methane/nitrogen separation. Nano Res. 2022, 15, 7695 10.1007/s12274-022-4352-0.

[ref79] Yang W.; Wang J.-X.; Yu B.; Li B.; Wang H.; Jiang J. A Robust Hydrogen-Bonded Organic Framework with 7-Fold Interpenetration Nets and High Permanent Microporosity. Cryst. Growth Des. 2022, 22 (3), 1817–1823. 10.1021/acs.cgd.1c01382.

[ref80] Yin Q.; Lü J.; Li H.-F.; Liu T.-F.; Cao R. Robust Microporous Porphyrin-Based Hydrogen-Bonded Organic Framework for Highly Selective Separation of C2 Hydrocarbons versus Methane. Cryst. Growth Des. 2019, 19 (7), 4157–4161. 10.1021/acs.cgd.9b00628.

[ref81] Domínguez-González R.; Rojas-León I.; Martínez-Ahumada E.; Martínez-Otero D.; Lara-García H. A.; Balmaseda-Era J.; Ibarra I. A.; Percástegui E. G.; Jancik V. UNAM-1: a robust CuI and CuII containing 3D-hydrogen-bonded framework with permanent porosity and reversible SO2 sorption. J. Mater. Chem. A 2019, 7 (47), 26812–26817. 10.1039/C9TA07834G.

[ref82] Li P.; He Y.; Arman H. D.; Krishna R.; Wang H.; Weng L.; Chen B. A microporous six-fold interpenetrated hydrogen-bonded organic framework for highly selective separation of C_2_H_4_/C_2_H_6_. Chem. Commun. 2014, 50 (86), 13081–13084. 10.1039/C4CC05506C. 25223376

[ref83] Li P.; He Y.; Zhao Y.; Weng L.; Wang H.; Krishna R.; Wu H.; Zhou W.; O’Keeffe M.; Han Y.; Chen B. A Rod-Packing Microporous Hydrogen-Bonded Organic Framework for Highly Selective Separation of C_2_H_2_/CO_2_ at Room Temperature. Angew. Chem., Int. Ed. 2014, 54 (2), 574–577. 10.1002/anie.201410077. 25394888

[ref84] Wang L.; Yang L.; Gong L.; Krishna R.; Gao Z.; Tao Y.; Yin W.; Xu Z.; Luo F. Constructing redox-active microporous hydrogen-bonded organic framework by imide-functionalization: Photochromism, electrochromism, and selective adsorption of C_2_H_2_ over CO_2_. Chem. Eng. J. 2020, 383, 123117 10.1016/j.cej.2019.123117.

[ref85] Wang H.; Li B.; Wu H.; Hu T.-L.; Yao Z.; Zhou W.; Xiang S.; Chen B. A Flexible Microporous Hydrogen-Bonded Organic Framework for Gas Sorption and Separation. J. Am. Chem. Soc. 2015, 137 (31), 9963–9970. 10.1021/jacs.5b05644. 26214340

[ref86] Yin Q.; Li Y.-L.; Li L.; Lü J.; Liu T.-F.; Cao R. Novel Hierarchical Meso-Microporous Hydrogen-Bonded Organic Framework for Selective Separation of Acetylene and Ethylene versus Methane. ACS Appl. Mater. Interfaces 2019, 11 (19), 17823–17827. 10.1021/acsami.9b03696. 31009575

[ref87] Yu B.; Geng S.; Wang H.; Zhou W.; Zhang Z.; Chen B.; Jiang J. A Solid Transformation into Carboxyl Dimers Based on a Robust Hydrogen-Bonded Organic Framework for Propyne/Propylene Separation. Angew. Chem., Int. Ed. 2021, 60 (49), 25942–25948. 10.1002/anie.202110057. 34499385

[ref88] Gao J.; Cai Y.; Qian X.; Liu P.; Wu H.; Zhou W.; Liu D.-X.; Li L.; Lin R.-B.; Chen B. A Microporous Hydrogen-Bonded Organic Framework for the Efficient Capture and Purification of Propylene. Angew. Chem., Int. Ed. 2021, 60 (37), 20400–20406. 10.1002/anie.202106665. 34219344

[ref89] Yang Y.; Li L.; Lin R.-B.; Ye Y.; Yao Z.; Yang L.; Xiang F.; Chen S.; Zhang Z.; Xiang S.; Chen B. Ethylene/ethane separation in a stable hydrogen-bonded organic framework through a gating mechanism. Nat. Chem. 2021, 13 (10), 933–939. 10.1038/s41557-021-00740-z. 34239085

[ref90] Chen Y.; Yang Y.; Wang Y.; Xiong Q.; Yang J.; Xiang S.; Li L.; Li J.; Zhang Z.; Chen B. Ultramicroporous Hydrogen-Bonded Organic Framework Material with a Thermoregulatory Gating Effect for Record Propylene Separation. J. Am. Chem. Soc. 2022, 144 (37), 17033–17040. 10.1021/jacs.2c06585. 36069372

[ref91] Yang Y.; Zhang H.; Yuan Z.; Wang J.-Q.; Xiang F.; Chen L.; Wei F.; Xiang S.; Chen B.; Zhang Z. An Ultramicroporous Hydrogen-Bonded Organic Framework Exhibiting High C_2_H_2_/CO_2_ Separation. Angew. Chem., Int. Ed. 2022, 61 (43), e202207579 10.1002/anie.202213957. 35833470

[ref92] Zhang X.; Wang J.-X.; Li L.; Pei J.; Krishna R.; Wu H.; Zhou W.; Qian G.; Chen B.; Li B. A Rod-Packing Hydrogen-Bonded Organic Framework with Suitable Pore Confinement for Benchmark Ethane/Ethylene Separation. Angew. Chem., Int. Ed. 2021, 60 (18), 10304–10310. 10.1002/anie.202100342. 33630416

[ref93] Liu J.; Miao J.; Ullah S.; Zhou K.; Yu L.; Wang H.; Wang Y.; Thonhauser T.; Li J. A Water-Resistant Hydrogen-Bonded Organic Framework for Ethane/Ethylene Separation in Humid Environments. ACS Mater. Lett. 2022, 4 (6), 1227–1232. 10.1021/acsmaterialslett.2c00370.

[ref94] Liang J.; Xing S.; Brandt P.; Nuhnen A.; Schlüsener C.; Sun Y.; Janiak C. A chemically stable cucurbit[6]uril-based hydrogen-bonded organic framework for potential SO_2_/CO_2_ separation. J. Mater. Chem. A 2020, 8 (38), 19799–19804. 10.1039/D0TA07457H.

[ref95] Wang J.-X.; Pei J.; Gu X.-W.; Lin Y.-X.; Li B.; Qian G. Efficient CO_2_/CO separation in a stable microporous hydrogen-bonded organic framework. Chem. Commun. 2021, 57 (78), 10051–10054. 10.1039/D1CC03438C. 34505863

[ref96] Feng S.; Shang Y.; Wang Z.; Kang Z.; Wang R.; Jiang J.; Fan L.; Fan W.; Liu Z.; Kong G.; Feng Y.; Hu S.; Guo H.; Sun D. Fabrication of a Hydrogen-Bonded Organic Framework Membrane through Solution Processing for Pressure-Regulated Gas Separation. Angew. Chem., Int. Ed. 2020, 59 (10), 3840–3845. 10.1002/anie.201914548. 31833627

[ref97] Ding X.; Liu Z.; Zhang Y.; Ye G.; Jia J.; Chen J. Binary Solvent Regulated Architecture of Ultra-Microporous Hydrogen-Bonded Organic Frameworks with Tunable Polarization for Highly-Selective Gas Separation. Angew. Chem., Int. Ed. 2022, 61 (13), e202116483 10.1002/anie.202116483. 35023611

[ref98] Chen C.; Guan H.; Li H.; Zhou Y.; Huang Y.; Wei W.; Hong M.; Wu M. A Noncovalent π-Stacked Porous Organic Molecular Framework for Selective Separation of Aromatics and Cyclic Aliphatics. Angew. Chem., Int. Ed. 2022, 61 (24), e202201646 10.1002/anie.202201646. 35352465

[ref99] Liu Y.; Wu H.; Guo L.; Zhou W.; Zhang Z.; Yang Q.; Yang Y.; Ren Q.; Bao Z. Hydrogen-Bonded Metal-Nucleobase Frameworks for Efficient Separation of Xenon and Krypton. Angew. Chem., Int. Ed. 2022, 61 (11), e202117609 10.1002/anie.202117609. 34989467

[ref100] Gong L.; Ye Y.; Liu Y.; Li Y.; Bao Z.; Xiang S.; Zhang Z.; Chen B. A Microporous Hydrogen-Bonded Organic Framework for Efficient Xe/Kr Separation. ACS Appl. Mater. Interfaces 2022, 14 (17), 19623–19628. 10.1021/acsami.2c04746. 35465666

[ref101] Li T.; Liu B.-T.; Fang Z.-B.; Yin Q.; Wang R.; Liu T.-F. Integrating active C3N4 moieties in hydrogen-bonded organic frameworks for efficient photocatalysis. J. Mater. Chem. A 2021, 9 (8), 4687–4691. 10.1039/D1TA00100K.

[ref102] Lin T.; Sun Y.; Tian C.; Wang D.; Hou L.; Ye F.; Zhao S. A silk-like hydrogen-bonded organic framework functionalized membrane with intrinsic catalytic activity for nonmetallic reduction of 4-nitrophenol. Chem. Eng. J. 2022, 441, 136092 10.1016/j.cej.2022.136092.

[ref103] Feng L.; Yuan Y.; Yan B.; Feng T.; Jian Y.; Zhang J.; Sun W.; Lin K.; Luo G.; Wang N. Halogen hydrogen-bonded organic framework (XHOF) constructed by singlet open-shell diradical for efficient photoreduction of U(VI). Nat. Commun. 2022, 13 (1), 1389 10.1038/s41467-022-29107-9. 35296676 PMC8927584

[ref104] Zhang A.-A.; Si D.; Huang H.; Xie L.; Fang Z.-B.; Liu T.-F.; Cao R. Partial Metalation of Porphyrin Moieties in Hydrogen-Bonded Organic Frameworks Provides Enhanced CO_2_ Photoreduction Activity. Angew. Chem., Int. Ed. 2022, 61 (28), e202203955 10.1002/anie.202203955. 35441462

[ref105] Wang Y.-R.; Liu M.; Gao G.-K.; Yang Y.-L.; Yang R.-X.; Ding H.-M.; Chen Y.; Li S.-L.; Lan Y.-Q. Implanting Numerous Hydrogen-Bonding Networks in a Cu-Porphyrin-Based Nanosheet to Boost CH_4_ Selectivity in Neutral-Media CO_2_ Electroreduction. Angew. Chem., Int. Ed. 2021, 60 (40), 21952–21958. 10.1002/anie.202108388. 34387026

[ref106] Yu B.; Li L.; Liu S.; Wang H.; Liu H.; Lin C.; Liu C.; Wu H.; Zhou W.; Li X.; Wang T.; Chen B.; Jiang J. Robust Biological Hydrogen-Bonded Organic Framework with Post-Functionalized Rhenium(I) Sites for Efficient Heterogeneous Visible-Light-Driven CO_2_ Reduction. Angew. Chem., Int. Ed. 2021, 60 (16), 8983–8989. 10.1002/anie.202016710. 33496055

[ref107] Yu B.; Meng T.; Ding X.; Liu X.; Wang H.; Chen B.; Zheng T.; Li W.; Zeng Q.; Jiang J. Hydrogen-Bonded Organic Framework Ultrathin Nanosheets for Efficient Visible-Light Photocatalytic CO_2_ Reduction. Angew. Chem., Int. Ed. 2022, 61 (43), e202211482 10.1002/anie.202211482. 36068668

[ref108] Han B.; Wang H.; Wang C.; Wu H.; Zhou W.; Chen B.; Jiang J. Postsynthetic Metalation of a Robust Hydrogen-Bonded Organic Framework for Heterogeneous Catalysis. J. Am. Chem. Soc. 2019, 141 (22), 8737–8740. 10.1021/jacs.9b03766. 31117661 PMC7928070

[ref109] Song Q.; Xu D.; David Wang W.; Fang J.; Sun X.; Li F.; Li B.; Kou J.; Zhu H.; Dong Z. Ru clusters confined in Hydrogen-bonded organic frameworks for homogeneous catalytic hydrogenation of N-heterocyclic compounds with heterogeneous recyclability. J. Catal. 2022, 406, 19–27. 10.1016/j.jcat.2021.09.027.

[ref110] Zhang N.; Yin Q.; Guo S.; Chen K.-K.; Liu T.-F.; Wang P.; Zhang Z.-M.; Lu T.-B. Hot-electron leading-out strategy for constructing photostable HOF catalysts with outstanding H_2_ evolution activity. Appl. Catal. B: Environ. 2021, 296, 120337 10.1016/j.apcatb.2021.120337.

[ref111] Aitchison C. M.; Kane C. M.; McMahon D. P.; Spackman P. R.; Pulido A.; Wang X.; Wilbraham L.; Chen L.; Clowes R.; Zwijnenburg M. A.; Sprick R. S.; Little M. A.; Day G. M.; Cooper A. I. Photocatalytic proton reduction by a computationally identified, molecular hydrogen-bonded framework. J. Mater. Chem. A 2020, 8 (15), 7158–7170. 10.1039/D0TA00219D.

[ref112] Zhang A.-A.; Li Y.-L.; Fang Z.-B.; Xie L.; Cao R.; Liu Y.; Liu T.-F. Facile Preparation of Hydrogen-Bonded Organic Framework/Cu_2_O Heterostructure Films via Electrophoretic Deposition for Efficient CO_2_ Photoreduction. ACS Appl. Mater. Interfaces 2022, 14 (18), 21050–21058. 10.1021/acsami.2c02917. 35476406

[ref113] Zhang H.; Yu D.; Liu S.; Liu C.; Liu Z.; Ren J.; Qu X. NIR-II Hydrogen-Bonded Organic Frameworks (HOFs) Used for Target-Specific Amyloid-β Photooxygenation in an Alzheimer’s Disease Model. Angew. Chem., Int. Ed. 2022, 61 (2), e202109068 10.1002/anie.202109068. 34735035

[ref114] He X.-T.; Luo Y.-H.; Hong D.-L.; Chen F.-H.; Zheng Z.-Y.; Wang C.; Wang J.-Y.; Chen C.; Sun B.-W. Atomically Thin Nanoribbons by Exfoliation of Hydrogen-Bonded Organic Frameworks for Drug Delivery. ACS Appl. Nano Mater. 2019, 2 (4), 2437–2445. 10.1021/acsanm.9b00303.

[ref115] Liu B.-T.; Pan X.-H.; Nie D.-Y.; Hu X.-J.; Liu E.-P.; Liu T.-F. Ionic Hydrogen-Bonded Organic Frameworks for Ion-Responsive Antimicrobial Membranes. Adv. Mater. 2020, 32 (48), 2005912 10.1002/adma.202005912. 33124716

[ref116] Liu B.-T.; Pan X.-H.; Zhang D.-Y.; Wang R.; Chen J.-Y.; Fang H.-R.; Liu T.-F. Construction of Function-Oriented Core-Shell Nanostructures in Hydrogen-Bonded Organic Frameworks for Near-Infrared-Responsive Bacterial Inhibition. Angew. Chem., Int. Ed. 2021, 60 (49), 25701–25707. 10.1002/anie.202110028. 34477299

[ref117] Tang J.; Liu J.; Zheng Q.; Li W.; Sheng J.; Mao L.; Wang M. In-Situ Encapsulation of Protein into Nanoscale Hydrogen-Bonded Organic Frameworks for Intracellular Biocatalysis. Angew. Chem., Int. Ed. 2021, 60 (41), 22315–22321. 10.1002/anie.202105634. 34382314

[ref118] Wied P.; Carraro F.; Bolivar J. M.; Doonan C. J.; Falcaro P.; Nidetzky B. Combining a Genetically Engineered Oxidase with Hydrogen-Bonded Organic Frameworks (HOFs) for Highly Efficient Biocomposites. Angew. Chem., Int. Ed. 2022, 61 (16), e202117345 10.1002/anie.202117345. PMC9305891 35038217

[ref119] Chen G.; Huang S.; Shen Y.; Kou X.; Ma X.; Huang S.; Tong Q.; Ma K.; Chen W.; Wang P.; Shen J.; Zhu F.; Ouyang G. Protein-directed, hydrogen-bonded biohybrid framework. Chem. 2021, 7 (10), 2722–2742. 10.1016/j.chempr.2021.07.003.

[ref120] Chen G.; Tong L.; Huang S.; Huang S.; Zhu F.; Ouyang G. Hydrogen-bonded organic framework biomimetic entrapment allowing non-native biocatalytic activity in enzyme. Nat. Commun. 2022, 13 (1), 4816 10.1038/s41467-022-32454-2. 35974100 PMC9381776

[ref121] Tang Z.; Li X.; Tong L.; Yang H.; Wu J.; Zhang X.; Song T.; Huang S.; Zhu F.; Chen G.; Ouyang G. A Biocatalytic Cascade in an Ultrastable Mesoporous Hydrogen-Bonded Organic Framework for Point-of-Care Biosensing. Angew. Chem., Int. Ed. 2021, 60 (44), 23608–23613. 10.1002/anie.202110351. 34459532

[ref122] Huang W.; Yuan H.; Yang H.; Tong L.; Gao R.; Kou X.; Wang J.; Ma X.; Huang S.; Zhu F.; Chen G.; Ouyang G. Photodynamic Hydrogen-Bonded Biohybrid Framework: A Photobiocatalytic Cascade Nanoreactor for Accelerating Diabetic Wound Therapy. JACS Au 2022, 2 (9), 2048–2058. 10.1021/jacsau.2c00321. 36186550 PMC9516711

[ref123] Yu D.; Zhang H.; Liu Z.; Liu C.; Du X.; Ren J.; Qu X. Hydrogen-Bonded Organic Framework (HOF)-Based Single-Neural Stem Cell Encapsulation and Transplantation to Remodel Impaired Neural Networks. Angew. Chem., Int. Ed. 2022, 61 (28), e202201485 10.1002/anie.202201485. 35385196

[ref124] Chen Q.; Zhang T.; Chen X.; Liang M.; Zhao H.; Yuan P.; Han Y.; Li C.-P.; Hao J.; Xue P. Tunable Fluorescence in Two-Component Hydrogen-Bonded Organic Frameworks Based on Energy Transfer. ACS Appl. Mater. Interfaces 2022, 14 (21), 24509–24517. 10.1021/acsami.2c05897. 35588507

[ref125] Xu X.; Yan B. Base-Tuning HOF-Based Host-Guest Ultralong Organic Phosphorescence Systems with Phosphorescent Thermochromism Using for Information Security and Thermometer. Adv. Opt. Mater. 2022, 10 (11), 2200451 10.1002/adom.202200451.

[ref126] Cai S.; An Z.; Huang W. Recent Advances in Luminescent Hydrogen-Bonded Organic Frameworks: Structures, Photophysical Properties, Applications. Adv. Funct. Mater. 2022, 32 (41), 2207145 10.1002/adfm.202207145.

[ref127] Lu Y.; Yu K.; Yin Q.; Liu J.; Han X.; Zhao D.; Liu T.; Li C. Embedding red-emitting dyes in robust hydrogen-bonded organic framework for application in warm white light-emitting diodes. Microporous Mesoporous Mater. 2022, 331, 111673 10.1016/j.micromeso.2021.111673.

[ref128] Liu B.-T.; Liu E.-P.; Sa R.-J.; Liu T.-F. Crystalline Hydrogen-Bonded Organic Chains Achieving Ultralong Phosphorescence via Triplet-Triplet Energy Transfer. Adv. Opt. Mater. 2020, 8 (12), 2000281 10.1002/adom.202000281.

[ref129] Zhang X.; Ren G.; He Z.; Yang W.; Li H.; Wang Y.; Pan Q.; Shi X. Luminescent Detection of Cr(VI) and Mn(VII) Based on a Stable Supramolecular Organic Framework. Cryst. Growth Des. 2020, 20 (10), 6888–6895. 10.1021/acs.cgd.0c00941.

[ref130] Feng J.-f.; Yan X.-Y.; Ji Z.-Y.; Liu T.-F.; Cao R. Fabrication of Lanthanide-Functionalized Hydrogen-Bonded Organic Framework Films for Ratiometric Temperature Sensing by Electrophoretic Deposition. ACS Appl. Mater. Interfaces 2020, 12 (26), 29854–29860. 10.1021/acsami.0c08354. 32483962

[ref131] Guo G.; Wang D.; Zheng X.; Bi X.; Liu S.; Sun L.; Zhao Y. Construction of tetraphenylethylene-based fluorescent hydrogen-bonded organic frameworks for detection of explosives. Dyes Pigments 2022, 197, 109881 10.1016/j.dyepig.2021.109881.

[ref132] Sun Z.; Li Y.; Chen L.; Jing X.; Xie Z. Fluorescent Hydrogen-Bonded Organic Framework for Sensing of Aromatic Compounds. Cryst. Growth Des. 2015, 15 (2), 542–545. 10.1021/cg501652r.

[ref133] Gomez E.; Suzuki Y.; Hisaki I.; Moreno M.; Douhal A. Spectroscopy and dynamics of a HOF and its molecular units: remarkable vapor acid sensing. J. Mater. Chem. C 2019, 7 (35), 10818–10832. 10.1039/C9TC03830B.

[ref134] Yu T.; Ou D.; Yang Z.; Huang Q.; Mao Z.; Chen J.; Zhang Y.; Liu S.; Xu J.; Bryce M. R.; Chi Z. The HOF structures of nitrotetraphenylethene derivatives provide new insights into the nature of AIE and a way to design mechanoluminescent materials. Chem. Sci. 2017, 8 (2), 1163–1168. 10.1039/C6SC03177C. 28616138 PMC5460603

[ref135] Huang Q.; Li W.; Yang Z.; Zhao J.; Li Y.; Mao Z.; Yang Z.; Liu S.; Zhang Y.; Chi Z. Achieving Bright Mechanoluminescence in a Hydrogen-Bonded Organic Framework by Polar Molecular Rotor Incorporation. CCS Chemistry 2022, 4 (5), 1643–1653. 10.31635/ccschem.021.202100968.

[ref136] Lv Y.; Li D.; Ren A.; Xiong Z.; Yao Y.; Cai K.; Xiang S.; Zhang Z.; Zhao Y. S. Hydrogen-Bonded Organic Framework Microlasers with Conformation-Induced Color-Tunable Output. ACS Appl. Mater. Interfaces 2021, 13 (24), 28662–28667. 10.1021/acsami.1c06312. 34114811

[ref137] Wang H.; Bao Z.; Wu H.; Lin R.-B.; Zhou W.; Hu T.-L.; Li B.; Zhao J. C.-G.; Chen B. Two solvent-induced porous hydrogen-bonded organic frameworks: solvent effects on structures and functionalities. Chem. Commun. 2017, 53 (81), 11150–11153. 10.1039/C7CC06187K. 28871296

[ref138] Huang Q.; Li W.; Mao Z.; Qu L.; Li Y.; Zhang H.; Yu T.; Yang Z.; Zhao J.; Zhang Y.; Aldred M. P.; Chi Z. An exceptionally flexible hydrogen-bonded organic framework with large-scale void regulation and adaptive guest accommodation abilities. Nat. Commun. 2019, 10 (1), 3074 10.1038/s41467-019-10575-5. 31300644 PMC6625987

[ref139] Huang Q.; Li W.; Mao Z.; Zhang H.; Li Y.; Ma D.; Wu H.; Zhao J.; Yang Z.; Zhang Y.; Gong L.; Aldred M. P.; Chi Z. Dynamic molecular weaving in a two-dimensional hydrogen-bonded organic framework. Chem. 2021, 7 (5), 1321–1332. 10.1016/j.chempr.2021.02.017.

[ref140] Jiang H.; Xie L.; Duan Z.; Lin K.; He Q.; Lynch V. M.; Sessler J. L.; Wang H. Fluorescent Supramolecular Organic Frameworks Constructed by Amidinium-Carboxylate Salt Bridges. Chem. Eur. J. 2021, 27 (60), 15006–15012. 10.1002/chem.202102296. 34288158

[ref141] Shi Y.; Wang S.; Tao W.; Guo J.; Xie S.; Ding Y.; Xu G.; Chen C.; Sun X.; Zhang Z.; He Z.; Wei P.; Tang B. Z. Multiple yet switchable hydrogen-bonded organic frameworks with white-light emission. Nat. Commun. 2022, 13 (1), 1882 10.1038/s41467-022-29565-1. 35388019 PMC8987099

[ref142] Han Y.; Zhang T.; Chen X.; Chen Q.; Hao J.; Song W.; Zeng Y.; Xue P. Guest-Regulated Luminescence and Force-Stimuli Response of a Hydrogen-Bonded Organic Framework. ACS Appl. Mater. Interfaces 2021, 13 (27), 32270–32277. 10.1021/acsami.1c08316. 34197080

[ref143] Xia G.; Jiang Z.; Shen S.; Liang K.; Shao Q.; Cong Z.; Wang H. Reversible Specific Vapoluminescence Behavior in Pure Organic Crystals through Hydrogen-Bonding Docking Strategy. Adv. Opt. Mater. 2019, 7 (8), 1801549 10.1002/adom.201801549.

[ref144] Shi Y.; Ding Y.; Tao W.; Wei P. Solvent-Triggered Fast and Visible Switching between Cage- and Channel-Type Hydrogen-Bonded Organic Frameworks. ACS Appl. Mater. Interfaces 2022, 14 (31), 36071–36078. 10.1021/acsami.2c11800. 35904893

[ref145] Wang C.; Wang Y.; Kirlikovali K. O.; Ma K.; Zhou Y.; Li P.; Farha O. K. Ultrafine Silver Nanoparticle Encapsulated Porous Molecular Traps for Discriminative Photoelectrochemical Detection of Mustard Gas Simulants by Synergistic Size-Exclusion and Site-Specific Recognition. Adv. Mater. 2022, 34 (35), 2202287 10.1002/adma.202202287. 35790037

[ref146] Wang Y.; Liu D.; Yin J.; Shang Y.; Du J.; Kang Z.; Wang R.; Chen Y.; Sun D.; Jiang J. An ultrafast responsive NO2 gas sensor based on a hydrogen-bonded organic framework material. Chem. Commun. 2020, 56 (5), 703–706. 10.1039/C9CC09171H. 31845686

[ref147] Zhou M.-Y.; Wang H.-Y.; Wang Z.-S.; Zhang X.-W.; Feng X.; Gao L.-Y.; Lian Z.-C.; Lin R.-B.; Zhou D.-D. Single-crystal superprotonic conductivity in an interpenetrated hydrogen-bonded quadruplex framework. Chem. Commun. 2022, 58 (6), 771–774. 10.1039/D1CC06004J. 34889324

[ref148] Xu X.-Q.; Cao L.-H.; Yang Y.; Zhao F.; Bai X.-T.; Zang S.-Q. Hybrid Nafion Membranes of Ionic Hydrogen-Bonded Organic Framework Materials for Proton Conduction and PEMFC Applications. ACS Appl. Mater. Interfaces 2021, 13 (47), 56566–56574. 10.1021/acsami.1c15748. 34787996

[ref149] Hao B.-B.; Wang X.-X.; Zhang C.-X.; Wang Q. Two Hydrogen-Bonded Organic Frameworks with Imidazole Encapsulation: Synthesis and Proton Conductivity. Cryst. Growth Des. 2021, 21 (7), 3908–3915. 10.1021/acs.cgd.1c00214.

[ref150] Wang Q.-X.; Guo Z.-C.; Qin Y.; Wang X.; Li G. High Proton Conduction in Three Highly Water-Stable Hydrogen-Bonded Ferrocene-Based Phenyl Carboxylate Frameworks. Inorg. Chem. 2021, 60 (24), 19278–19286. 10.1021/acs.inorgchem.1c03093. 34860499

[ref151] Wang Y.; Zhang M.; Yang Q.; Yin J.; Liu D.; Shang Y.; Kang Z.; Wang R.; Sun D.; Jiang J. Single-crystal-to-single-crystal transformation and proton conductivity of three hydrogen-bonded organic frameworks. Chem. Commun. 2020, 56 (99), 15529–15532. 10.1039/D0CC05402J. 33220663

[ref152] Chand S.; Pal S. C.; Pal A.; Ye Y.; Lin Q.; Zhang Z.; Xiang S.; Das M. C. Metalo Hydrogen-Bonded Organic Frameworks (MHOFs) as New Class of Crystalline Materials for Protonic Conduction. Chem. Eur. J. 2019, 25 (7), 1691–1695. 10.1002/chem.201805177. 30462360

[ref153] Qin Y.; Gao T.-l.; Xie W.-P.; Li Z.; Li G. Ultrahigh Proton Conduction in Two Highly Stable Ferrocenyl Carboxylate Frameworks. ACS Appl. Mater. Interfaces 2019, 11 (34), 31018–31027. 10.1021/acsami.9b11056. 31381293

[ref154] Karmakar A.; Illathvalappil R.; Anothumakkool B.; Sen A.; Samanta P.; Desai A. V.; Kurungot S.; Ghosh S. K. Hydrogen-Bonded Organic Frameworks (HOFs): A New Class of Porous Crystalline Proton-Conducting Materials. Angew. Chem., Int. Ed. 2016, 55 (36), 10667–10671. 10.1002/anie.201604534. 27464784

[ref155] Wang Y.; Yin J.; Liu D.; Gao C.; Kang Z.; Wang R.; Sun D.; Jiang J. Guest-tuned proton conductivity of a porphyrinylphosphonate-based hydrogen-bonded organic framework. J. Mater. Chem. A 2021, 9 (5), 2683–2688. 10.1039/D0TA07207A.

[ref156] Feng J.-f.; Liu T.-F.; Cao R. An Electrochromic Hydrogen-Bonded Organic Framework Film. Angew. Chem., Int. Ed. 2020, 59 (50), 22392–22396. 10.1002/anie.202006926. 32885555

[ref157] Kirlikovali K. O.; Goswami S.; Mian M. R.; Krzyaniak M. D.; Wasielewski M. R.; Hupp J. T.; Li P.; Farha O. K. An Electrically Conductive Tetrathiafulvalene-Based Hydrogen-Bonded Organic Framework. ACS Mater. Lett. 2022, 4, 128–135. 10.1021/acsmaterialslett.1c00628.

[ref158] Khanpour M.; Deng W.-Z.; Fang Z.-B.; Li Y.-L.; Yin Q.; Zhang A.-A.; Rouhani F.; Morsali A.; Liu T.-F. Radiochromic Hydrogen-Bonded Organic Frameworks for X-ray Detection. Chem. Eur. J. 2021, 27 (42), 10957–10965. 10.1002/chem.202101061. 33884685

[ref159] Chen T.-H.; Popov I.; Kaveevivitchai W.; Chuang Y.-C.; Chen Y.-S.; Daugulis O.; Jacobson A. J.; Miljanić O. Š. Thermally robust and porous noncovalent organic framework with high affinity for fluorocarbons and CFCs. Nat. Commun. 2014, 5 (1), 5131 10.1038/ncomms6131. 25307413

[ref160] Li P.; He Y.; Guang J.; Weng L.; Zhao J. C.-G.; Xiang S.; Chen B. A Homochiral Microporous Hydrogen-Bonded Organic Framework for Highly Enantioselective Separation of Secondary Alcohols. J. Am. Chem. Soc. 2014, 136 (2), 547–549. 10.1021/ja4129795. 24392725

[ref161] Li Y.; Tang S.; Yusov A.; Rose J.; Borrfors A. N.; Hu C. T.; Ward M. D. Hydrogen-bonded frameworks for molecular structure determination. Nat. Commun. 2019, 10 (1), 4477 10.1038/s41467-019-12453-6. 31578331 PMC6775153

[ref162] Takeda T.; Ozawa M.; Akutagawa T. Jumping Crystal of a Hydrogen-Bonded Organic Framework Induced by the Collective Molecular Motion of a Twisted π System. Angew. Chem., Int. Ed. 2019, 58 (30), 10345–10352. 10.1002/anie.201905075. 31106500

[ref163] Chen L.; Zhang B.; Chen L.; Liu H.; Hu Y.; Qiao S. Hydrogen-bonded organic frameworks: design, applications, and prospects. Materials Advances 2022, 3 (9), 3680–3708. 10.1039/D1MA01173A.

[ref164] Kaushik A.; Marvaniya K.; Kulkarni Y.; Bhatt D.; Bhatt J.; Mane M.; Suresh E.; Tothadi S.; Patel K.; Kushwaha S. Large-area self-standing thin film of porous hydrogen-bonded organic framework for efficient uranium extraction from seawater. Chem. 2022, 8 (10), 2749–2765. 10.1016/j.chempr.2022.07.009.

[ref165] Kaushik A.; Marvaniya K.; Kulkarni Y.; Bhatt D.; Bhatt J.; Mane M.; Suresh E.; Tothadi S.; Patel K.; Kushwaha S. Large-area self-standing thin film of porous hydrogen-bonded organic framework for efficient uranium extraction from seawater. Chem. 2022, 8, 2749 10.1016/j.chempr.2022.07.009.

